# Evaluation of Blood-Based Biomarkers for Clinical Discrimination of Asymptomatic Carotid Artery Stenosis Patients with Ulcerated Plaques

**DOI:** 10.2174/0113892029361154251029052509

**Published:** 2026-04-15

**Authors:** Ceyda Colakoglu Bergel, Atif Yolgosteren, Isil Ezgi Eryilmaz, Unal Egeli, Can Kosukcu, Guven Ozkaya, Basak Erdemli Gursel, Murat Bicer, Gulsah Cecener, Efe Ozoglu, Emre Sarandol, Mustafa Tok

**Affiliations:** 1 Bursa Uludag University, Graduate School of Medical Sciences, Medical Biology Department, Gorukle, Bursa, Turkiye;; 2 Bursa Uludag University, Faculty of Medicine, Cardiovascular Surgery Department, Gorukle, Bursa, Turkiye;; 3 Bursa Uludag University, Faculty of Medicine, Medical Biology Department, Gorukle, Bursa, Turkiye;; 4 Hacettepe University, Institute of Child Health, Pediatric Metabolism Department, Ankara, Turkiye;; 5 Bursa Uludag University, Faculty of Medicine, Bioistatistic Department, Gorukle, Bursa, Turkiye;; 6 Bursa Uludag University, Faculty of Medicine, Radiology Department, Gorukle, Bursa, Turkiye;; 7 Bursa Uludag University, Faculty of Medicine, Medical Biochemistry Department, Gorukle, Bursa, Turkiye

**Keywords:** Asymptomatic carotid artery stenosis, biomarker profiling, plaque biology, RNA-Seq, smooth plaque, ulcerated plaque

## Abstract

**Background:**

Although analysis of plaque composition using noninvasive imaging helps identify plaques prone to rupture, simultaneous assessment of factors involved in ulceration associated with plaque rupture, with imaging of carotid plaque characteristics, is essential to identify high-risk groups in asymptomatic carotid stenosis (ACS).

**Aims:**

The current study aimed to investigate blood-based biomarkers for detecting plaque ulceration in asymptomatic carotid stenosis (ACS) patients by analyzing plaque material and surface morphology using RNA sequencing (RNA-Seq).

**Materials and Methods:**

After RNA isolation, RNA-Seq and analyzing were performed using the Ion AmpliSeq Transcriptome Human Gene Expression Panel and IPA. Expression levels were analyzed to validate IPA results using RT-qPCR and ELISA.

**Results & Discussion:**

Based on IPA results, the genes that can be used in this discrimination were determined to be involved in the pathways as follows: (i) actin skeletal system, (ii) integrin, and (iii) ABRA signaling. Then, the determined fold changes were validated in RT-qPCR. Based on results, *CHI3L2, IL-6*, and *CCL24* gene expression levels were determined to be -2.91-, -1.3-, and -3.66- fold downregulated, and *FHL5* gene expression levels were determined to be 1,61- fold upregulated (*p*<0.05). After validation in blood material, only the *FHL5* expression level was determined to be upregulated in both plaque and blood materials.

**Conclusion:**

This is the first study to report that blood *FHL5* levels may help discriminate high-risk groups of asymptomatic carotid stenosis (ACS) patients with ulcerated plaques, based on plaque surface morphology and biological characteristics. Thus, this study provides the first evidence supporting the biomarker potential of *FHL5* gene expression levels in the blood of ACS patients with ulcerated plaques.

## 1 INTRODUCTION

Cardiovascular diseases are classified as coronary artery disease (CAD), cerebrovascular disease, peripheral artery disease (PAD), and aortic atherosclerosis [1]. Atherosclerosis is a chronic and multifactorial disease with several potential factors contributing to the formation of plaques, not limited to abnormal lipid accumulation [2]. Carotid atherosclerosis (CAS) is the narrowing of the carotid arteries caused by plaque. CAS is a manifestation of cerebrovascular atherosclerosis and is a disease that increases the risk of fatal and nonfatal myocardial infarction. CAS is also one of the leading causes of cerebrovascular strokes [3].

CAS patients are classified as “symptomatic” and “asymptomatic” based on their symptoms. Asymptomatic carotid stenosis (ACS) is defined as the presence of 50% or more stenosis of the proximal internal carotid artery at the level of the bifurcation in individuals with no history of ischemic stroke in the ipsilateral carotid region within the last six months [4]. Although various methods are used for the treatment of ACS, including carotid endarterectomy (CEA), carotid artery stenting, and best medical treatment (BMT), treatment approaches are controversial [5]. In most cases, the diagnosis of ACS patients can be determined incidentally on axial imaging after listening to the carotid bruit. Thus, treatment is a major problem due to the lack of symptoms. Additionally, only a small minority of ACS patients benefit from carotid endarterectomy (CEA) or stenting [6]. Moreover, the use of imaging methods for the diagnosis and screening of ACS has several disadvantages, including their high cost and their inappropriateness for universal screening, and there is still a need to define the high-risk group in ACS who may benefit from interventional treatments [7].

Carotid plaques are classified as smooth, irregular, and ulcerated based on their surface morphology. Carotid plaque ulceration is defined as irregularity, cracking, or erosion of the plaque surface, with a portion of the interior of the plaque in direct contact with circulating blood, which is associated with the consequences of vulnerable plaque [8]. Additionally, ulcerated plaques are considered a marker of previous plaque rupture. Noninvasive imaging assessment of carotid plaque ulceration is limited to patients with ≥50% carotid stenosis, with two-thirds of ulcerations occurring in low-grade carotid arteries, and little is known about the relationship between carotid plaque characteristics and plaque ulceration in these patients [9]. Although analysis of plaque composition using noninvasive imaging helps identify plaques prone to rupture, simultaneous assessment of factors involved in ulceration progression associated with plaque rupture, with imaging of carotid plaque characteristics, is essential to identify high-risk groups. In the current study, RNA-based biomarkers were found to be significant by evaluating plaque transcriptome levels in ACS patients, whose plaque surface morphologies, ulcerated or smooth, were determined by imaging methods, and the significant plaque-based biomarkers were then validated in blood samples of the ACS patients. Although there are various biomarker studies to assess plaque's biological character [10-12], this is the first study in which blood-based biomarker determination was performed by evaluating the biological character of plaque in the discrimination of ulcerated or smooth plaque in ACS patients.

## 2 MATERIALS AND METHODS

### 2.1 The Study Cohort

A total of 24 ACS patients with stenosis above 70%, as determined by plaque elasticity and morphology using Doppler ultrasonography, underwent carotid endarterectomy (CAE) and were enrolled in the current study. The plaque surface morphology was assessed by an expert (B.E.G.) and further confirmed through post-surgical examination by A.Y., M.B., and M.T. Plaques were then classified as either ulcerated or smooth. Since the risk of cardiovascular disease, including atherosclerosis, is higher in individuals over the age of 60, and well-developed atherosclerotic plaques are typically diagnosed only after this age, patients aged 60 and over were considered the high-risk group [13, 14]. Therefore, to identify potential blood-based biomarkers in high-risk ACS patients, only individuals aged 60 and over were included in the study.

Thus, the inclusion criteria for patients were: (1) age 60 years or older, (2) stenosis > 70% according to the North American Carotid Endarterectomy Trial (NASCET) criteria, and (3) no history of ischemic stroke in the ipsilateral carotid region within the past six months. The exclusion criteria were: (1) age under 60 years, (2) stenosis < 70% according to NASCET criteria, and (3) a history of ischemic stroke within the past six months, malignancies, or other chronic illnesses that could influence gene expression profiles (Fig. **1**).

As a discovery group, 10 ACS patients, including 5 (50%) with ulcerated plaques, were included for RNA-Seq analysis. Candidate biomarker genes identified in the plaque materials using RNA-Seq were subsequently validated by RT-qPCR. For the validation group, 24 plaques were selected, including 11 (45.8%) ulcerated and 13 (54.2%) smooth plaques. The candidate biomarker genes validated in these plaque materials were then further verified in 19 blood samples from ACS patients, comprising 10 (52.6%) with ulcerated and 9 (47.4%) with smooth plaques, to assess their potential for discriminating plaque ulceration. All participants provided written informed consent for the use of isolated RNA samples in the RNA-Seq analysis conducted at the Department of Medical Biology, Bursa Uludag University. This study was approved by the Uludag University Medical Ethics Committee (2021-6/38) and complied with the ethical standards of the Helsinki Declaration.

### 2.2 RNA-Seq Analysis

The effect of sample size and minimum detectable effect size was calculated based on the literature for RNA-Seq analysis [15]. In different scenarios with minimum detectable effect sizes ranging from 1.20 to 2.0, the calculation was carried out with 80% power. Effect sizes are shown as fold change (FC). If the cut-off value for FC is selected as 2.0, the sample size for each group corresponds to ~6 at 80% power [15]. However, in our study, each RNA-Seq group comprised 5 samples, corresponding to ~80% power for > 2.0 FC.

Total RNA was isolated from plaque and blood samples of ACS patients, grouped according to plaque surface morphology, using the RNeasy Lipid Tissue Mini Kit (Qiagen, Hilden, Germany). For blood samples, red blood cells were lysed by adding Red Blood Lysis (RBL) buffer. Briefly, RBL buffer was added to the blood at a 1:10 ratio and incubated at 4°C for 15 minutes. After incubation, the samples were centrifuged at 600 × g for 10 minutes, washed with PBS, and Qiazol was added directly to the pellets. Samples were stored at –20°C and shaken vigorously in the lysis buffer for 1 minute. Chloroform was then added, and the samples were centrifuged at 12,000 × g for 15 minutes at 4°C. The supernatant was collected, mixed with 70% ethanol, and transferred to a spin column. After centrifugation and washing, 35 µL of RNase-free water was added directly to the column membrane, and the samples were centrifuged at 8,000 × g for 1 minute.

For RNA isolation from plaque materials, tissue samples were lysed using a TissueLyser (Qiagen, Hilden, Germany) with beads in 700 µL of Qiazol, and the same protocol was subsequently applied. Finally, all RNA samples were stored at –80°C.

RNA qualities and concentrations were analyzed using the Agilent 2100 (Agilent Technologies, Santa Clara, California, USA). Samples with DV200 scores above 70% were included in the RNA-Seq analysis as follows. The mRNA libraries were prepared using the Ion AmpliSeq™ Chef-Ready Kit (Thermo Fisher Scientific, Massachusetts, USA) for the Ion AmpliSeq Transcriptome Human Gene Expression Panel on an Ion GeneStudio™ S5 System (Thermo Fisher Scientific, Massachusetts, USA). First, cDNA conversion was performed using 10 ng of each RNA sample to prepare 100 pM cDNA libraries by performing 31 cycles with the Ion Chef Instrument (Thermo Fisher Scientific, Massachusetts, USA). Then, library samples were diluted to 80 pM, and chip loading was performed using the Ion Chef Instrument (Thermo Fisher Scientific, Massachusetts, USA). Finally, sequencing was performed with an Ion 540™ Chip on the Ion GeneStudio™ S5 System (Thermo Fisher Scientific, Massachusetts, USA).

After sequencing, data were processed using the QIAGEN CLC Genomics Workbench. Pairwise comparisons of two groups, ACS patients with ulcerated or smooth plaque, were performed using the QIAGEN Ingenuity Pathway Analysis (IPA). A *p*-value correction was performed using the false discovery rate (FDR) method to eliminate false-positive genes. The adjusted cut-off *p*-value for statistically significant genes was set at 0.01, and genes below this value were considered statistically significant. Downregulated and upregulated genes were determined by fold changes (FC) as follows: >2.0 FC and corrected *p* ˂ 0.01.

### 2.3 Gene Expression Analysis

Based on the RNA-Seq and IPA results, the expression analysis for 4 genes, including *Interleukin-6 (IL-6)*, *Chitinase 3-like 2 (CHI3L2)*, *Four and a Half LIM Domain 5 (FHL5)*, and *C-C motif ligand 24 (CCL24)*, was performed using StepOnePlus Real-Time PCR System (Applied Biosystems, Waltham, Massachusetts, USA) with Promega SYBR Green Master Mix (Madison, Wisconsin, USA). Gene expression levels were normalized to the *GAPDH* expression status. The 5'→3' primer sequences for the genes were as follows: *FHL5*: F: CATTTTGTGACCAGCTATGGCA and R: AGGCTACACACTTGTTGGCA; *CHI3L2:* F: GGTGGATTCTTCTACATCACGC and R: TCAGCACAGTGAAATGAGTGTT; *IL-6:* F: CCTTCGG TCCAGTTGCCTTC and R: TGAGATGCCGTCGAGGATG; *CCL24:* F: CTGCAAGGACCCGAGCTATT and R: GATGATGTGGTGGGCACAGA; *GAPDH:* F: CTGCC ACCCAGAAGACTGTG and R: TGGGTGTCGCTGTTGAAGCT. Then, the statistical analysis of the gene expression results was performed according to the 2^−ΔΔCT^ relative expression method using the QIAGEN GeneGlobe Data Analysis Center, which is available online at https://geneglobe.qiagen.com/tr/analyze.

### 2.4 Evaluation of Serum Protein Levels in Patients with Ulcerated Plaque

Serum samples obtained from yellow-capped tubes were analyzed for LPL, MMP9, APOC1, APOC2, IL-6, and Neutrophil Gelatinase-Associated Lipocalin (NGAL) (Bioassay Technology Laboratory, Shanghai, China) levels. For this purpose, 96-well plates were shaken at room temperature for 1 hour, samples coated with protein antibodies were washed, and Avidin-HRP conjugated reagents were added. The reaction was stopped after the substrate was added to the incubated samples. Then, measurements were made at 450 nm using a multifunctional microplate reader. Sample amounts were measured using the appropriate standard curve, and all tests were duplicated.

### 2.5 Statistical Analysis

In the current study, the chi-square (χ2) test was used to compare the categorical clinical characteristics of ACS patients and the ulcerated *vs*. smooth plaques. The descriptive statistics were presented as mean ± standard deviation or frequency and percentage. Differences were considered statistically significant at a *p*<0.05. The statistical tests were performed using IBM SPSS 29.0.2.0 (IBM Corp. Released 2023. IBM SPSS Statistics for Windows, Version 29.0.2.0 Armonk, NY: IBM Corp.)

## 3 RESULTS

### 3.1 The Clinical Characteristics of ACS Patients with Ulcerated and Smooth Plaques

For the discovery group, a total of 10 patients with ACS were included in this study. Of these, 50% (n=5) were male and 50% (n=5) were female. According to the anamnesis, 50% (n=5) of the patients had ulcerated plaque. The mean age was 68.2 ± 6.0 years. Additionally, 80% (n=8) of the patients had hypertension, 70% (n=7) had diabetes, and 10% (n=1) had hyperlipidemia. Moreover, 10% (n=1) of the patients had peripheral artery disease, and 20% (n=2) had coronary artery disease. The presence of left and right stenosis was equal distributed (n=5).

For the validation group, a total of 24 plaque samples were included in the current study. Among the patients, 58.3% (n=14) were male and 41.7% (n=10) were female. Based on the anamnesis, 45.8% (n=11) of ACS patients had ulcerated plaque. The mean age was 66.7 ± 8.5 years. Also, 70.8% (n=17) of the patients had hypertension, 54.2% (n=13) had diabetes, and 12.5% (n=3) had hyperlipidemia, 25.0% (n=6) had peripheral artery disease, and 29.2% (n=7) had coronary artery disease. The left-sided stenosis of the patients was 54.2% (n=13), and the right-sided was 45.8% (n=11).

For the blood-based validation group, a total of 19 patients with ACS were included. Among them, 57.9% (n=11) were male, and 42.1% (n=8) were female. The mean age was 66.7 ± 7.6 years. Additionally, 63.2% (n=12) of the patients had hypertension, 47.4% (n=9) had diabetes, and 15.8% (n=3) had hyperlipidemia. Moreover, 21.1% (n=4) of the patients had peripheral artery disease, and 26.3% (n=5) had coronary artery disease. The left-sided stenosis of the patients was 68.4% (n=13), and the right-sided was 31.6% (n=6). The clinical characteristics of the patients are shown in Table **1**.

### 3.2 Evaluations of Expression Profiles and Pathway Analysis

IPA analysis was performed to identify genes with differential expression levels in discriminating ACS patients with ulcerated *versus* smooth plaques. In total, 463 DEGs were detected between the two groups, as listed in Supplementary Material **1**. Based on the IPA analysis results, we found that the top canonical pathways and regulator effect networks are commonly regulated. Thus, we evaluated the “Top Ready Molecules” section by considering the canonical pathways and the top regulators listed in Supplementary Material **1**. According to the IPA results, various signaling pathways, including cell motility, cell death and survival, and cell proliferation, were re-regulated in the ulcerated plaque samples of ACS patients. The genes that could be used for discrimination were determined to be involved in the following pathways: (i) actin cytoskeleton signaling, (ii) integrin signaling, and (iii) ABRA signaling. All activated or suppressed pathways are indicated in Fig. **2A-C**. The fold-change (FC) values of the gene expression levels are presented in Fig. **2D-E**.

When the canonical pathway and top regulator sections were examined, it was determined that there were significant regulations of the immune system and the cytoskeleton signaling pathways (Actin Cytoskeleton Signaling, Integrin Signaling, and ABRA Signaling Pathway). Then, we filtered the genes related to these signaling pathways from the ready molecules with FC >2.0-fold and *p*˂0.01, and the final DEGs were detected as *CHI3L2, IL-6, CCL24*, and *FHL5* between the two groups (Fig. **2D**). Then, the determined expression status of the genes was validated by RT-qPCR using 24 plaque materials. Based on the results, *CHI3L2, IL-6*, and *CCL24* gene expression levels were determined to be 2.91-, 1.3-, and 3.66-fold downregulated, and *FHL5* gene expression level was determined to be 1.61-fold upregulated (*p*<0.05) (Fig. **2E**), respectively. Additionally, the activating (orange) and repressing (blue) interactions in (i) actin skeletal system signaling, (ii) integrin signaling, and (iii) ABRA signaling pathways are indicated in Fig. **3A**, **B**, and **C**, respectively.

Based on the molecular interaction analysis, actin/integrin polymerization and cytoskeletal remodeling are mainly activated in the ulcerated plaques compared to the smooth plaques. Interestingly, the results also show that actin and integrin polymerization and cytoskeletal remodeling are activated, as illustrated in Fig. **3C**. Moreover, cell motility and cytoskeletal remodeling pathways are activated in the ulcerated plaques, suggesting that these genes and their cumulative interactions alter cell behavior by re-regulating the cytoskeletal system in ulcerated plaques compared to smooth plaques.

### 3.3 Validation of DEGs in Blood Materials

Statistically significant candidate gene expression levels from IPA analyses were investigated in patients' blood materials to identify blood-based RNA biomarkers in ACS patients.

After the plaque-based validation, *FHL5, CHI3L2, IL-6,* and *CCL24* gene expression levels were subsequently validated in the blood materials of ACS patients. These genes were upregulated by 2.75-, 3.50-, 2.26-, and 4.84-fold, respectively (*p*>0.05) in the blood samples of ACS patients with ulcerated plaques compared to those with smooth ones. Consequently, only the *FHL5* gene expression level was shown to be upregulated in both blood and ulcerated plaque materials of ACS patients with ulcerated plaques (Fig. **4A**).

### 3.4 Analysis of Serum Levels of CAS-associated Proteins

In our study, we primarily focused on discovering biomarkers at the RNA level for ACS patients with ulcerated plaques. To validate and compare our findings with the previously established biomarkers for ACS, we measured the protein levels of LPL, MMP9, APOC1, APOC2, IL-6, and NGAL with ELISA, which are known to have a strong association with atherosclerosis in the literature [16-21]. These measured proteins were selected based on their well-known associations with our disease model in the literature previously. Since RNA-Seq analysis did not detect any significant regulations at the mRNA expression of these proteins (Supp. Material 1), we investigated the serum levels of the proteins with well-known associations to determine whether the detected mRNAs after RNA-Seq in our study could serve as more potential biomarkers than the serum levels of these proteins. This approach allowed us to assess whether post-transcriptional mechanisms or alternative regulatory pathways of these genes contributed to their presence at the protein level despite the lack of detectable mRNA levels of these proteins in RNA-Seq analysis. Interestingly, the results showed that serum levels of the indicated proteins were not statistically significant in the discrimination of ulcerated plaques from the smooth ones (Fig. **4B**).

## 4 DISCUSSION

The current study aimed to investigate a blood-based biomarker for detecting plaque ulceration by analyzing plaque materials based on surface morphology using RNA-Seq in ACS patients. The results suggested that FHL5 gene expression might serve as a biomarker candidate for differentiating ACS patients with ulcerated plaques from those with smooth plaques.

IPA results showed that (i) actin cytoskeleton signaling, (ii) integrin signaling, and (iii) ABRA signaling were deregulated in ACS patients with ulcerated plaques compared to those with smooth plaques. While genes including CHI3L2, IL-6, and CCL24 were downregulated, FHL5 expression was upregulated in ACS patients with ulcerated plaques (Fig. **2D**).

Integrins promote actin assembly by recruiting molecules that directly activate the actin polymerization machinery or physically link it to cell adhesion sites, serving as major mediators of vascular cell adhesion and migration [22]. The link between integrins and actin is known to regulate the site of cell migration and plays an important role in adhesion organization and development [23]. ABRA is a muscle-specific actin-binding protein that can stimulate transcription *via* a pathway involving RhoA and actin polymerization. Studies have shown that ABRA plays a role in human skeletal muscle hypertrophy and atrophy, and that ABRA activates Rho signaling, affecting actin dynamics and cytoskeletal integrity [24]. Chemokine signals regulate migration by activating leukocyte integrins and the actin remodeling machinery, which are critical for leukocyte adhesion and mobility across vascular barriers. More importantly, chemokines have been shown to trigger integrin activation and actin remodeling events that direct lymphocyte migration across vascular barriers [25]. Therefore, our study, in which actin, integrin, and ABRA signaling were identified as primary regulators in ulcerated plaques, suggests that immune and skeletal system signaling may be associated with leukocyte migration and enhanced immunity.

CHI3L2/YKL-39 is a chitinase-like protein belonging to the glycoside hydrolase 18 family that plays a role in autoimmune response by inducing the production of pro- and anti-inflammatory cytokines and tissue remodeling [26]. A study has shown that YKL-40, another chitinase-like protein, is a marker for activated macrophages within atherosclerotic plaques and is expressed in different macrophage subpopulations in atherosclerotic lesions. Moreover, in a recent study, mRNA expression of *YKL-40* was found in macrophages in early atherosclerotic lesions [27, 28]. Interestingly, this chitinase-like protein has been shown to promote smooth muscle cell migration and neovascularization in plaques *via* AKT and ERK signaling pathways and to reduce collagen content in late-stage atherosclerotic lesions of the carotid arteries [29]. In advanced plaques, collagen is the major component of the ECM, comprising up to 60% of the total protein content, and a dynamic imbalance between collagen synthesis and degradation has been shown to weaken the fibrous cap, leading to a decrease in collagen content [30]. Therefore, it is thought that downregulation of this gene in ulcerated plaque may trigger the rupture-prone structure of the plaque due to decreased collagen content.

IL-6 plays a role in immunological processes by binding to the membrane-bound IL-6 receptor (IL-6R) and regulating inflammatory signaling. Basal levels of IL-6 are known to regulate lipid homeostasis and vascular remodeling in atherosclerotic conditions. A recent study stated that increased levels of IL-6 secreted from lipid-laden macrophages may play a protective role by controlling cholesterol accumulation [31]. Our data suggest that decreased *IL-6* levels in ACS patients with ulcerated plaques may be due to a decreased protective effect on lipid-laden macrophages. Another gene, *CCL24*, encodes a pro-inflammatory chemokine belonging to the CC chemokine ligand family, expressed in various chronic diseases [32]. CCL24 plays a role in the progression of VSMC calcification, migration, and proliferation [33]. The downregulation of *CCL24* also contributes to the recruitment of neutrophils and macrophages, suggesting that downregulation of *CCL24* expression might be associated with increased macrophage accumulation due to enhanced immune response in ACS patients with ulcerated plaques [34].

FHL5 is a member of the cofactor family with a transcriptional activation function that is expressed in coordination with an activator of the cAMP-responsive element modulator (CREM). In a previous study, FHL5 was shown to increase the risk of vascular disease by regulating vascular remodeling through its transcriptional regulatory role, and its overexpression promoted vascular calcification [35]. Studies have also indicated that FHL5 colocalizes with RUNX2 near intimal calcium deposits, which are associated with arterial calcification. It has been reported that FHL5 promotes a shift toward the SMC osteogenic phenotypic state, increasing the risk of various cardiovascular events through vascular remodeling [35]. Moreover, Dong *et al.* reported that FHL5 was among the top ten upregulated genes when comparing microarray data from macroscopically intact tissue with atheroma plaques in 64 samples collected from 32 hypertensive patients who had suffered CAE [36]. Interestingly, a previous study found that FHL5 expression was 1.23-fold downregulated in atheromatous plaques compared to intact tissues in 23 carotid atherosclerotic plaques and 11 carotid intact tissues. However, after comparing public datasets, that study specified that FHL5 was not among the top six DEGs [37]. Additionally, our study showed that *FHL5* was upregulated in both plaque and blood samples of ACS patients with ulcerated plaques compared to those with smooth plaques, suggesting that *FHL5* upregulation may contribute to arterial calcification and vascular remodeling. This conclusion is supported by the DEGs identified in our IPA analysis, including actin cytoskeleton signaling, integrin signaling, and ABRA signaling.

In conclusion, the study suggests that actin cytoskeleton, integrin, and ABRA signaling were deregulated in ACS patients with ulcerated plaques compared to those with smooth plaques, through dysregulation of the related genes *CHI3L2*, *IL-6*, *CCL24*, and *FHL5* during ulcerated plaque progression. Moreover, this is the first study to report that blood *FHL5* levels may be used to identify the risk group of ACS patients with ulcerated plaques based on plaque surface morphology and biology, considering that *FHL5* expression was higher in both plaque and blood samples of these patients. When assessing the biomarker potential of *FHL5* by comparing its serum levels with those of proteins strongly associated with atherosclerosis in the literature, we found that *FHL5* expression could be a more promising biomarker than these proteins. Therefore, *FHL5* gene expression may serve as a blood-based biomarker for discriminating ACS patients with ulcerated plaques. However, the utility of *FHL5* as a blood-based biomarker should be validated in larger populations.

## CONCLUSION

Consequently, the present study revealed that actin cytoskeleton, integrin, and ABRA signaling are significantly regulated in ACS patients with ulcerated plaques compared to those with smooth plaques, accompanied by dysregulation of the related genes *CHI3L2*, *IL-6*, *CCL24*, and *FHL5* during ulcerated plaque progression. Moreover, this is the first study to report that blood *FHL5* levels may be used to identify the high-risk group of ACS patients with ulcerated plaques based on plaque surface morphology and biology. Thus, this study provides the first evidence for the biomarker potential of gene expression levels in blood samples of ACS patients with ulcerated plaques. Since this is an unexplored area, our primary aim was to identify potential mRNA biomarker candidates. Therefore, as a limitation, we note that *FHL5* levels should be investigated in larger cohorts or through further protein-based *in vitro* studies to gain deeper insights into its relevance in ACS pathogenesis.

## Figures and Tables

**Fig. (1):**
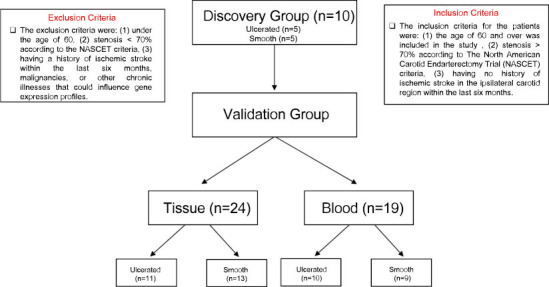
A diagram for inclusion and exclusion criteria of ACS patients and their materials enrolled in the current study.

**Fig. (2):**
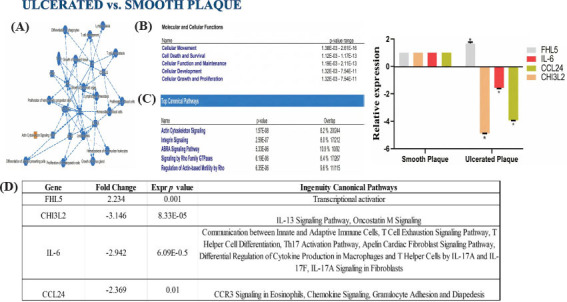
IPA analysis of RNA-Seq results in plaque materials for ulcerated-smooth discrimination. (**A**) Activated (orange) and repressed pathways (blue) in ulcerated plaque (**B**) and (**C**) Molecular/cellular functions of genes involved in this differentiation and the pathways they are involved in (**D**) Fold changes and canonical pathways of statistically significant genes involved in the pathways. (**E**) Validation of RNA-Seq results using RT-qPCR. Canonical pathway figures are generated using QIAGEN Ingenuity Pathway Analysis (IPA), QIAGEN Inc.

**Fig. (3):**
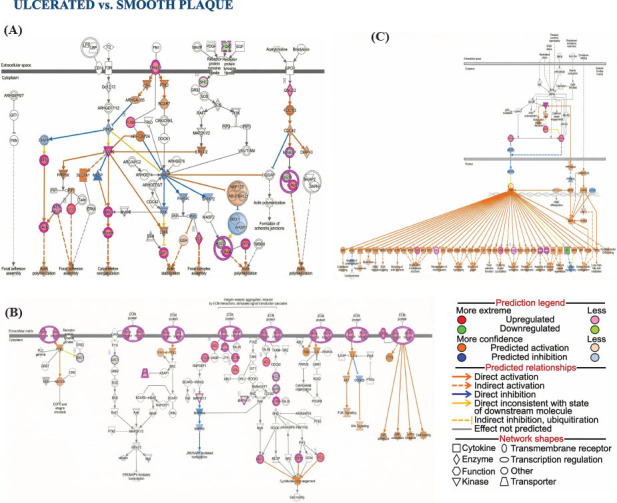
IPA analysis of RNA-Seq results in plaque materials for ulcerated *vs*. smooth plaque discrimination (**A**) actin skeletal system signaling (**B**) integrin signaling (**C**) ABRA signaling pathways. Canonical pathway figures are generated using QIAGEN Ingenuity Pathway Analysis (IPA), QIAGEN Inc.

**Fig. (4):**
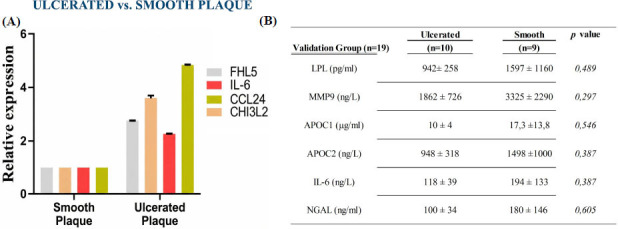
(**A**) Validation of statistically significant genes detected in plaques for the discrimination between ulcerated and smooth plaque (in blood material) (**B**) Serum protein levels for the discrimination between ulcerated *vs*. smooth plaque.

**Table 1:** Clinical characteristics of ACS patients for discovery and validation groups.

-	**Ulcerated**	**Smooth**	***p-*value**	-	**Ulcerated**	**Smooth**	***p-*value**	-	**Ulcerated**	**Smooth**	***p-*value**
**Discovery Group (n=10)**	**(n=5)**	**(n=5)**	**-**	**Validation Group (n=24) (for tissue)**	**(n=11)**	**(n=13)**	-	**Validation Group (n=19) (for blood)**	**(n=10)**	**(n=9)**	-
**Gender (n, %)**	**-**	**-**	**-**	**Gender (n, %)**	-	-	-	**Gender (n, %)**	-	-	-
Female (n=5)	60% (n=3)	40% (n=2)	*1*	Female (n=13)	63,6% (n=7)	46,2% (n=6)	*0,697*	Female (n=8)	40% (n=4)	44,4% (n=4)	*1*
Male (n=5)	40% (n=2)	60% (n=3)		Male (n=11)	36,4% (n=4)	53,8% (n=7)		Male (n=11)	60% (n=6)	55,6% (n=5)	
**Age (Mean ± SD)**	68,6 ± 4,3	67,4 ± 8,2	*0,781*	**Age (Mean ± SD)**	66 ± 6,7	67,3 ± 9,9	*0,719*	**Age (Mean ± SD)**	65,3 ± 6,5	68,3 ± 8,7	*0,403*
**Diabetes Mellitus**	80% (n=4)	60% (n=3)	*1*	**Diabetes Mellitus**	54,5% (n=6)	53,8% (n=7)	*0,973*	**Diabetes Mellitus**	50% (n=5)	44,4% (n=4)	*1*
**Hyperlipidemia**	(n=0)	20% (n=1)	*1*	**Hyperlipidemia**	9,1% (n=1)	15,4% (n=2)	*1*	**Hyperlipidemia**	10% (n=1)	22,2% (n=2)	*0,582*
**Hypertension**	100% (n=1)	60% (n=3)	*0,444*	**Hypertension**	81,8% (n=9)	61,5% (n=8)	*0,386*	**Hypertension**	80% (n=8)	44,4% (n=4)	*0,17*
**Coronary Artery Disease**	(n=0)	40% (n=2)	*0,444*	**Coronary Artery Disease**	9,1% (n=1)	46,2% (n=6)	*0,07*	**Coronary Artery Disease**	10% (n=1)	44,4% (n=4)	*0,141*
**Stenosis Side**	-	-	*1*	**Stenosis Side**	-	-	*0,392*	**Stenosis Side**	-	-	*1*
Right (n=5)	40% (n=2)	60% (n=3)		Right (n=11)	36,4% (n=4)	53,8% (n=7)		Right (n=6)	30% (n=3)	33,3% (n=3)	
Left (n=5)	60% (n=3)	40% (n=2)		Left (n=13)	63,6% (n=7)	46,2% (n=6)		Left (n=13)	70% (n=7)	66,7% (n=6)	
**Peripheral Artery Disease**	(n=0)	20% (n=1)	*1*	**Peripheral Artery Disease**	36,4% (n=4)	15,4% (n=2)	*0,357*	**Peripheral Artery Disease**	30% (n=3)	11,1% (n=1)	*0,582*

## Data Availability

Not applicable.

## LIST OF ABBREVIATIONS

ACS: Asymptomatic Carotid Stenosis
APOC1: Apolipoprotein C-1
APOC2: Apolipoprotein C-2
APOE: Apolipoprotein E
CAS: Carotid Artery Stenosis
CCL24: C-C Motif Ligand 24
CEA: Carotid Endarterectomy
CHI3L2: Chitinase 3-like 2
FHL5: Four and a Half LIM Domains 5
IL-6: Interleukin-6
IPA: Ingenuity Pathway Analysis
LPL: Lipoprotein Lipase
