# Supplementary material for: Evaluation of Blood-Based Biomarkers for Clinical Discrimination of Asymptomatic Carotid Artery Stenosis Patients with Ulcerated Plaques
Source: Curr Genomics. 2026 Apr 15;26(6):599–608. doi: 10.2174/0113892029361154251029052509 (PMC13154241; doi:10.2174/0113892029361154251029052509)
Supplement: Supplementary file 1 [file CG-26-6-599_SD1.pdf]

INGENUITY
PATHWAY ANALYSIS

Analysis Name: AU vs. AD 2023-08-09 11:13:36
Analysis Creation Date: 2023-08-09
Build version: exported
Content version: Not available.

Experiment Metadata

Name	Value

Analysis Settings

Reference set: Ingenuity Knowledge Base (Genes Only)
Relationship to include: Direct and Indirect
Includes Endogenous Chemicals
Optional Analyses: My Pathways My List

Filter Summary:
Consider only molecules and/or relationships where
(species = Human OR Mammal OR Rat OR Uncategorized OR Mouse) AND
(confidence = Experimentally Observed) AND
(tissues/cell lines = SK-MEL-2 OR Other Macrophages OR Th1 cells OR Astrocytes OR LOX IMVI OR Naive B cells OR RPMI-8266 OR Microglia OR Kidney Cancer Cell Lines OR Calvaria OR SN12C OR Cerebellum OR Activated Vd2 Gamma-delta T cells OR CD4+ T-lymphocytes OR Thymus OR Kidney OR BT-549 OR BDCA-1+ dendritic cells OR Testis OR RBL-2H3 OR Other Cell Line OR Crypt OR Natural

(c) 2000-2024 QIAGEN. All rights reserved.

T-regulatory cells OR Other Peripheral blood leukocytes OR NCI-H23 OR Cortical neurons OR Other Lymphoma Cell Lines OR NB4 OR A375 OR Small Intestine OR J-774A.1 OR A549-ATCC OR PC-3 OR HepG2 OR T lymphocytes OR Other Monocytes OR Trachea OR Ovarian Cancer Cell Lines OR Other Neuroblastoma Cell Lines OR Oocytes OR Adipose OR Other Granulocytes OR Blood platelets OR NK cells not otherwise specified OR HOP-92 OR MDA-MB-435 OR Other T lymphocytes OR Monocyte-derived macrophage OR Other Hepatoma Cell Lines OR Immature monocyte-derived dendritic cells OR Memory B cells OR Th17 cells OR HT29 OR Adipocytes OR Epithelial cells not otherwise specified OR Sciatic Nerve OR Bone marrow-derived dendritic cells OR Cos-7 cells OR NCI-H332M OR SF-268 OR Lymphoma Cell Lines OR Gray Matter OR Dorsal Root Ganglion OR SF-295 OR Liver OR Organ Systems not otherwise specified OR Breast Cancer Cell Lines OR Macrophages not otherwise specified OR Immune cells OR Bladder OR Eosinophils OR Memory T lymphocytes OR CAKI-1 OR MCF7 OR Colon Cancer Cell Lines not otherwise specified OR Other Breast Cancer Cell Lines OR NT2/D1 OR CNS Cell Lines not otherwise specified OR Mammary Gland OR Monocytes OR 3T3-L1 cells OR U251 OR Adrenal Gland OR Langerhans cells OR Hippocampus OR Effector memory RA+ cytotoxic T cells OR OVCAR-4 OR Hepatocytes OR OVCAR-8 OR Purkinje cells OR UACC-62 OR Neuroblastoma Cell Lines not otherwise specified OR Cervical cancer cell line OR BDCA-3+ dendritic cells OR Mononuclear leukocytes not otherwise specified OR Other Lymphocytes OR SW-620 OR HEL OR HOP-62 OR OVCAR-5 OR Ventricular Zone OR NCI-H522 OR Megakaryocytes OR Spinal Cord OR Immune cell lines OR BA/F3 OR Lymphoma Cell Lines not otherwise specified OR CD56bright NK cells OR Monocyte-derived dendritic cells not otherwise specified OR Vd1 Gamma-delta T cells OR HCT-116 OR Granule Cell Layer OR Lymphocytes not otherwise specified OR Uterus OR Bone marrow cells not otherwise specified OR Lens OR Intraepithelial T lymphocytes OR Pancreatic Cancer Cell Lines not otherwise specified OR Smooth muscle cells OR CNS Cell Lines OR Neurons not otherwise specified OR Melanocytes OR SW-480 OR PANC-1 OR Fibroblast cell lines not otherwise specified OR Teratocarcinoma Cell Lines not otherwise specified OR Swiss 3T3 cells OR Other Bone marrow cells OR Microvascular endothelial cells OR Brainstem OR Other Lung Cancer Cell Lines OR Naive helper T cells OR Tissues and Primary Cells not otherwise specified OR Salivary Gland OR Olfactory Bulb OR MDA-N OR Cells OR Other Nervous System OR Other NK cells OR Peripheral blood leukocytes not otherwise specified OR Cytotoxic T cells OR Choroid Plexus OR Stem cells OR LNCaP cells OR SK-MEL-28 OR T lymphocytes not otherwise specified OR Dendritic cells OR Nervous System OR Pheochromocytoma cell lines OR SF-539 OR Cartilage Tissue OR Medulla Oblongata OR B lymphocytes not otherwise specified OR Pro-B lymphocytes OR HMC-1 OR U87MG OR Heart OR Skin OR INS-1 OR K-562 OR Other Melanoma Cell Lines OR Ovarian Cancer Cell Lines not otherwise specified OR Beta islet cells OR Parietal Lobe OR KM-12 OR Kidney Cancer Cell Lines not otherwise specified OR Melanoma Cell Lines OR Osteosarcoma Cell Lines OR Peripheral blood monocytes OR Cardiomyocytes OR Other Leukemia Cell Lines OR Other Teratocarcinoma Cell Lines OR Other Cervical cancer cell line OR Brain OR IGROV1 OR Other Fibroblast cell lines OR Activated Vd1 Gamma-delta T cells OR Lymphocytes OR OVCAR-3 OR Central memory helper T cells OR U937 OR Plasma cells OR Chondrocytes OR Immune cell lines not otherwise specified OR Macrophage Cancer Cell Lines OR Hematopoietic progenitor cells OR HL-60 OR Forestomach OR Memory T lymphocytes not otherwise specified OR Other Smooth muscle cells

(c) 2000-2024 QIAGEN. All rights reserved.

OR Vd2 Gamma-delta T cells OR Th2 cells OR Striatum OR Other Dendritic cells OR Neuroblastoma Cell Lines OR DU-145 OR CD34+ cells OR M14 OR HeLa OR Neutrophils OR Hep3B OR Other Immune cell lines OR UO-31 OR Central memory cytotoxic T cells OR Stromal cells OR Large Intestine OR White Matter OR Monocytes not otherwise specified OR Prostate Cancer Cell Lines not otherwise specified OR Bone marrow cells OR H460 OR T47-D OR Cervical cancer cell line not otherwise specified OR Prostate Gland OR Peritoneal macrophages OR Other Macrophage Cancer Cell Lines OR Lymph node OR Placenta OR Kidney cell lines not otherwise specified OR Other Cells OR Hepatoma Cell Lines OR Corpus Callosum OR Effector memory helper T cells OR Pancreas OR Melanoma Cell Lines not otherwise specified OR Activated helper T cells OR Other Organ Systems OR Fibroblasts OR NIH/3T3 cells OR Other Monocyte-derived dendritic cells OR SR OR Other Neurons OR UACC-257 OR Other Endothelial cells OR Other Pheochromocytoma cell lines OR Thymocytes OR Esophagus OR MALME-3M OR Keratinocytes OR Other Myeloma Cell Lines OR Peripheral blood lymphocytes OR Lung Cancer Cell Lines OR Other Kidney Cancer Cell Lines OR MDA-MB-361 OR MDA-MB-231 OR PC-12 cells OR Cells not otherwise specified OR HCC-2998 OR NCI-H226 OR Ovary OR Osteosarcoma Cell Lines not otherwise specified OR Dendritic cells not otherwise specified OR Myeloma Cell Lines not otherwise specified OR Smooth muscle cells not otherwise specified OR Hepatoma Cell Lines not otherwise specified OR U2OS OR Skeletal Muscle OR Mesenchymal stem cells OR Pancreatic Cancer Cell Lines OR Granulosa cells OR Other Tissues and Primary Cells OR CCRF-CEM OR Stomach OR Thalamus OR Leukemia Cell Lines not otherwise specified OR THP-1 OR Other Memory T lymphocytes OR Activated CD56bright NK cells OR Nervous System not otherwise specified OR Amygdala OR Fibroblast cell lines OR Activated CD56dim NK cells OR Granule cells OR HuH7 OR Plasmacytoid dendritic cells OR HUVEC cells OR Monocyte-derived dendritic cells OR Min6 OR Retina OR Other Ovarian Cancer Cell Lines OR Jurkat OR RKO OR Immune cells not otherwise specified OR Other CNS Cell Lines OR Other B lymphocytes OR Granulocytes OR Hypothalamus OR Splenocytes OR HS 578T OR Caudate Nucleus OR Lung OR Cerebral Cortex OR NK cells OR Embryonic stem cells OR Effector T cells OR Other Kidney cell lines OR WEHI-231 OR Other Colon Cancer Cell Lines OR Other Epithelial cells OR Other Stem cells OR Other Mononuclear leukocytes OR NCI-ADR-RES OR Stem cells not otherwise specified OR Vascular smooth muscle cells OR Kidney cell lines OR RAW 264.7 OR B lymphocytes OR Bone marrow-derived macrophages OR SK-MEL-5 OR Pheochromocytoma cell lines not otherwise specified OR Pre-B lymphocytes OR Teratocarcinoma Cell Lines OR Cerebral Ventricles OR U266 OR 786-0 OR Other Immune cells OR Sertoli cells OR Leukemia Cell Lines OR ACHN OR Mononuclear leukocytes OR A498 OR J774 OR Cell Line not otherwise specified OR Pituitary Gland OR Effector memory cytotoxic T cells OR Endothelial cells not otherwise specified OR Nucleus Accumbens OR Osteoblasts OR P19 OR Breast Cancer Cell Lines not otherwise specified OR Myeloid dendritic cells OR MG-63 OR Macrophage Cancer Cell Lines not otherwise specified OR Substantia Nigra OR Granulocytes not otherwise specified OR Colon Cancer Cell Lines OR BT-474 OR SK-OV-3 OR Epithelial cells OR Mast cells OR Prostate Cancer Cell Lines OR RXF-393 OR Macrophages OR MDA-MB-468 OR Cornea OR Pyramidal neurons OR SK-N-SH OR Putamen OR Neurons OR CD56dim NK cells OR Epidermis OR Myeloma Cell Lines OR A2780 OR Lung Cancer Cell Lines not otherwise specified OR Endothelial cells OR Other Osteosarcoma Cell Lines OR Dermis OR MEF cells OR Smooth Muscle OR HCT-15 OR

(c) 2000-2024 QIAGEN. All rights reserved.

INGENUITY PATHWAY ANALYSIS

Other Pancreatic Cancer Cell Lines OR Spleen OR COLO205 OR Subventricular Zone OR Other Prostate Cancer Cell Lines OR Peripheral blood leukocytes OR Thyroid Gland OR Caco2 cells OR PBMCs OR Trigeminal Ganglion OR Mature monocyte-derived dendritic cells OR EKVX OR TK-10 OR 293 cells OR Murine NKT cells OR MOLT-4 OR Organ Systems OR SNB-75) AND
(data sources = An Open Access Database of Genome-wide Association Results OR BIND OR BioGRID OR Catalogue Of Somatic Mutations In Cancer (COSMIC) OR Chemical Carcinogenesis Research Information System (CCRIS) OR Clinical Genome Resource (ClinGen) OR ClinicalTrials.gov OR ClinVar OR Cognia OR DIP OR DrugBank OR Gene Ontology (GO) OR GVK Biosciences OR Hazardous Substances Data Bank (HSDB) OR HumanCyc OR Ingenuity Expert Findings OR Ingenuity ExpertAssist Findings OR IntAct OR Interactome studies OR MIPS OR miRBase OR miRecords OR Mouse Genome Database (MGD) OR Obesity Gene Map Database OR Online Mendelian Inheritance in Man (OMIM) OR TarBase OR TargetScan Human)

Top Canonical Pathways

Name	p-value	Overlap
**Actin Cytoskeleton Signaling**	1.57E-08	8.2 % 20/244
**Integrin Signaling**	2.59E-07	8.0 % 17/212
**ABRA Signaling Pathway**	5.32E-06	10.9 % 10/92
**Signaling by Rho Family GTPases**	6.19E-06	6.4 % 17/267
**Regulation of Actin-based Motility by Rho**	6.35E-06	9.6 % 11/115

Top Upstream Regulators

Upstream Regulators

Name	p-value	Predicted Activation

(c) 2000-2024 QIAGEN. All rights reserved.

INGENUITY PATHWAY ANALYSIS

**TNF**	2.54E-16	Inhibited
**dexamethasone**	9.09E-16	
**lipopolysaccharide**	1.47E-15	Inhibited
**TGFB1**	4.55E-14	
**beta-estradiol**	1.72E-13	

Causal Network

Name	p-value	Predicted Activation
**NEO2734**	3.91E-22	
**hydroxypropyl-beta-cyclodextrin**	1.15E-20	
**BTB 09588**	1.24E-19	Activated
**RDR 03785**	1.24E-19	Activated
**DFP 00129**	1.24E-19	Activated

Top Diseases and Bio Functions

Diseases and Disorders

Name	p-value range	# Molecules
**Cancer**	1.38E-03 - 2.66E-26	416
**Organismal Injury and Abnormalities**	1.41E-03 - 2.66E-26	423
**Endocrine System Disorders**	9.08E-04 - 1.83E-17	355
**Inflammatory Response**	1.23E-03 - 1.86E-16	181
**Immunological Disease**	1.38E-03 - 2.88E-15	201

(c) 2000-2024 QIAGEN. All rights reserved.

INGENUITY PATHWAY ANALYSIS

Molecular and Cellular Functions

Name	p-value range	# Molecules
**Cellular Movement**	1.38E-03 - 2.61E-16	159
**Cell Death and Survival**	1.12E-03 - 1.17E-13	182
**Cellular Function and Maintenance**	1.19E-03 - 2.11E-13	208
**Cellular Development**	1.32E-03 - 7.54E-11	211
**Cellular Growth and Proliferation**	1.32E-03 - 7.54E-11	206

Physiological System Development and Function

Name	p-value range	# Molecules
**Cardiovascular System Development and Function**	1.41E-03 - 1.27E-20	139
**Organismal Development**	1.41E-03 - 2.56E-19	226
**Hematological System Development and Function**	1.38E-03 - 3.41E-12	142
**Immune Cell Trafficking**	1.38E-03 - 3.41E-12	91
**Tissue Development**	1.41E-03 - 8.82E-11	202

Top Tox Functions

Assays: Clinical Chemistry and Hematology

(c) 2000-2024 QIAGEN. All rights reserved.

INGENUITY PATHWAY ANALYSIS

Name	p-value range	# Molecules
**Increased Levels of ALT**	1.33E-01 - 2.34E-03	4
**Decreased Levels of Albumin**	1.02E-01 - 4.42E-03	4
**Increased Levels of Albumin**	1.83E-02 - 1.83E-02	2
**Increased Levels of CRP**	3.51E-02 - 3.51E-02	1
**Increased Levels of Red Blood Cells**	5.69E-02 - 5.69E-02	5

Cardiotoxicity

Name	p-value range	# Molecules
**Cardiac Dysfunction**	5.91E-01 - 1.27E-05	31
**Heart Failure**	3.82E-01 - 1.85E-04	19
**Cardiac Congestive Cardiac Failure**	3.01E-03 - 1.89E-04	11
**Cardiac Necrosis/Cell Death**	2.21E-01 - 2.68E-04	14
**Cardiac Enlargement**	4.35E-01 - 4.01E-04	32

Hepatotoxicity

Name	p-value range	# Molecules
**Liver Hyperplasia/Hyperproliferation**	1.00E00 - 1.39E-04	164
**Liver Necrosis/Cell Death**	3.74E-01 - 5.50E-04	16
**Hepatocellular carcinoma**	1.00E00 - 9.80E-04	66
**Liver Inflammation/Hepatitis**	3.71E-01 - 2.83E-03	20
**Liver Cirrhosis**	5.67E-02 - 3.06E-03	15

(c) 2000-2024 QIAGEN. All rights reserved.

INGENUITY PATHWAY ANALYSIS

Nephrotoxicity

Name	p-value range	# Molecules
**Renal Inflammation**	5.52E-01 - 4.12E-05	24
**Renal Nephritis**	5.52E-01 - 4.12E-05	24
**Glomerular Injury**	1.00E00 - 2.09E-04	30
**Renal Necrosis/Cell Death**	4.23E-01 - 9.06E-04	24
**Nephrosis**	3.37E-01 - 9.46E-03	7

Top Regulator Effect Networks

ID	Regulators	Disease & Functions	Consistency Score
**1**	CD40LG,CHROMR,DUSP1,EGF,FOXA2,IFN Beta,IFNA2,IFNG (+17 more)	Cell movement of antigen presenting cells (+10 more)	46.667
**2**	ADAMTS12,BANF1,CD40LG,CHROMR,DUSP1,EGF,ESR1 (+31 more)	Cell movement of B lymphocytes (+12 more)	44.429
**3**	CHROMR,DUSP1,IFN Beta,IFNA2,Ige,IL1,IL17A,IL1A (+10 more)	Cell movement of leukemia cell lines (+11 more)	42.875
**4**	ADAMTS12,BANF1,CD40LG,IFNG,IL15,IL1A,IRF2BP2,IRF7 (+2 more)	Chemotaxis of myeloid cells (+7 more)	38.655
**5**	CSF,IFN Beta,Ige,IL15,KLF6,P38 MAPK,PF4,PTGS2,TLR2 (+3 more)	Chemotaxis of myeloid cells (+7 more)	34.064

Top Networks

ID	Associated Network Functions	Score

(c) 2000-2024 QIAGEN. All rights reserved.

**1**	Cancer, Cardiovascular Disease, Cell Cycle	52
**2**	Embryonic Development, Organismal Development, Tissue Morphology	43
**3**	Lipid Metabolism, Small Molecule Biochemistry, Vitamin and Mineral Metabolism	37
**4**	Infectious Diseases, Organismal Injury and Abnormalities, Cardiovascular System Development and Function	37
**5**	Post-Translational Modification, Cardiovascular Disease, Developmental Disorder	37

Top Tox Lists

Name	p-value	Overlap
**Hepatic Fibrosis**	1.55E-05	5.5 % 19/347
**Liver Necrosis/Cell Death**	7.00E-04	4.5 % 16/358
**Renal Necrosis/Cell Death**	9.72E-04	3.6 % 24/672
**LXR/RXR Activation**	1.57E-03	6.5 % 8/123
**Cardiac Hypertrophy**	1.58E-03	4.1 % 16/387

(c) 2000-2024 QIAGEN. All rights reserved.

Top My Lists

Top My Pathways

Top ML Disease Pathways

Name	p-value	Overlap
**Systolic dysfunction**	1.49E-05	19.4 % 6/31
**Immunodeficiency**	3.67E-05	16.7 % 6/36
**Nephritis**	1.41E-04	17.2 % 5/29
**Mixed hematopoietic and lymphoid cancer**	1.72E-04	12.8 % 6/47
**Lymphoproliferative malignancy**	1.72E-04	12.8 % 6/47

Top Analysis-Ready Molecules

Expr Log Ratio

Molecules	Expr. Value	Chart
**RERGL**	↑ 2.679	
**ID3**	↑ 2.655	
**CASQ2**	↑ 2.485	
**FHL5**	↑ 2.234	

(c) 2000-2024 QIAGEN. All rights reserved.

INGENUITY PATHWAY ANALYSIS

**SUSD5**	↑ 2.037	
**FRZB**	↑ 1.977	
**SH3BGR**	↑ 1.900	
**ECM2**	↑ 1.875	
**ST6GAL2**	↑ 1.838	
**LDOC1**	↑ 1.793	

Expr Log Ratio

Molecules	Expr. Value	Chart
**FAM157A/FAM157B**	↓ -3.900	
**CXCL13**	↓ -3.551	
**CHI3L2**	↓ -3.146	
**IL6**	↓ -2.942	
**CD79A**	↓ -2.792	
**CCL19**	↓ -2.604	
**LCE3E**	↓ -2.562	
**PF4V1**	↓ -2.294	
**MZB1**	↓ -2.235	
**CADM3**	↓ -2.130	

(c) 2000-2024 QIAGEN. All rights reserved.

© 2000-2024 QIAGEN. All rights reserved.

	Symbol	Entrez Ger	Ensembl/E	Expr p-valı	Expr p-valı	Expr False	Expr Fold (	Expr Log F	Expr Inten:
1	A2M	alpha-2-ma	ENSG000(	0.0323	1	0.422	1.74	0.799	178.13
2	ACER2	alkaline ce	ENSG000(	0.048	1	0.465	1.653	0.725	11.213
3	ACTA2	actin alpha	ENSG000(	0.0171	1	0.342	2.203	1.139	1608.613
4	ACTC1	actin alpha	ENSG000(	0.0107	1	0.288	2.676	1.42	12.202
5	ACTN4	actinin alpl	ENSG000(	0.0247	1	0.38	1.863	0.897	109.371
6	ADH5	alcohol del	ENSG000(	0.0445	1	0.458	1.628	0.703	87.162
7	ADH1B	alcohol del	ENSG000(	0.0209	1	0.36	2.645	1.403	142.279
8	ADI1	acireducto	ENSG000(	0.00691	1	0.26	1.828	0.87	29.335
9	AKTIP	AKT intera	ENSG000(	0.00189	1	0.166	2.121	1.085	17.292
10	ALOX5	arachidona	ENSG000(	0.0164	1	0.339	-1.895	-0.923	22.327
11	ANGPTL4	angiopoiet	ENSG000(	0.00128	1	0.148	-3.988	-1.996	16.122
12	ANKRD37	ankyrin rep	ENSG000(	0.0017	1	0.164	2.078	1.055	17.966
13	ANO1	anoctamin	ENSG000(	0.0049	1	0.232	2.087	1.061	15.507
14	ANO6	anoctamin	ENSG000(	0.0276	1	0.394	1.57	0.651	18.867
15	ANTXR1	ANTXR ce	ENSG000(	0.035	1	0.427	2.051	1.036	80.427
16	ANXA7	annexin A7	ENSG000(	0.0459	1	0.46	1.527	0.611	84.713
17	AOC3	amine oxid	ENSG000(	0.0015	1	0.159	2.427	1.279	37.094
18	AP3S1	adaptor rel	ENSG000(	0.0445	1	0.458	1.489	0.574	34.745
19	API5	apoptosis i	ENSG000(	0.0177	1	0.345	1.566	0.647	14.971
20	ARF4	ADP ribosy	ENSG000(	0.0089	1	0.273	1.762	0.817	91.842
21	ARHGAP4	Rho GTPa	ENSG000(	0.00745	1	0.264	-1.824	-0.867	13.548
22	ARMC1	armadillo r	ENSG000(	0.00963	1	0.281	1.682	0.75	17.57
23	ARMCX1	armadillo r	ENSG000(	0.0475	1	0.465	1.441	0.527	35.302
24	ARPC5L	actin relate	ENSG000(	0.00276	1	0.188	-1.977	-0.983	18.092
25	ASPN	asporin	ENSG000(	0.0414	1	0.45	2.329	1.219	149.671
26	ATAD1	ATPase fa	ENSG000(	0.0112	1	0.295	1.633	0.708	15.206
27	ATL3	atlastin GT	ENSG000(	0.0218	1	0.366	1.713	0.776	28.867
28	B3GALNT:	beta-1,3-N	ENSG000(	0.0249	1	0.382	1.553	0.635	10.369
29	BBX	BBX high r	ENSG000(	0.0125	1	0.311	1.748	0.806	15.341
30	BORCS5	BLOC-1 re	ENSG000(	0.0431	1	0.458	1.551	0.633	14.626
31	BRMS1	BRMS1 tra	ENSG000(	0.0242	1	0.377	-1.714	-0.777	26.559
32	C15orf48	chromosor	ENSG000(	0.0198	1	0.356	-2.461	-1.299	41.683
33	C1GALT1(	C1GALT1	ENSG000(	0.0358	1	0.428	1.566	0.647	23.694
34	C1orf122	chromosor	ENSG000(	0.0498	1	0.47	-1.499	-0.584	19.284
35	C1orf198	chromosor	ENSG000(	0.0186	1	0.352	1.745	0.803	27.992
36	C1QTNF2	C1q and T	ENSG000(	0.0144	1	0.328	1.917	0.939	10.121
37	C8orf88	chromosor	ENSG000(	0.0297	1	0.405	1.904	0.929	32.424
38	CADM3	cell adhesi	ENSG000(	0.000237	1	0.0746	-4.378	-2.13	18.589
39	CALD1	caldesmon	ENSG000(	0.0453	1	0.459	1.891	0.919	128.471
40	CAP1	cyclase as	ENSG000(	0.0306	1	0.41	1.652	0.724	64.149
41	CAP2	cyclase as	ENSG000(	0.026	1	0.388	1.829	0.871	28.589
42	CASC3	CASC3 ex	ENSG000(	0.0251	1	0.384	1.605	0.682	20.943
43	CASQ2	calsequest	ENSG000(	2.21E-05	0.6	0.0223	5.599	2.485	10.547
44	CAV2	caveolin 2	ENSG000(	0.0133	1	0.318	2.058	1.041	37.581
45	CAVIN3	caveolae a	ENSG000(	0.0487	1	0.468	1.64	0.713	222.451
46	CBR1	carbonyl re	ENSG000(	0.0363	1	0.43	1.52	0.604	14.664
47	CCDC146	coiled-coil	ENSG000(	0.0128	1	0.314	1.847	0.885	10.751
48	CCL2	C-C motif (	ENSG000(	0.0141	1	0.324	-2.217	-1.149	1002.436
49	CCL19	C-C motif (	ENSG000(	0.00089	1	0.132	-6.078	-2.604	435.817
50	CCL24	C-C motif (	ENSG000(	0.0194	1	0.354	-2.369	-1.244	23.066

**51**	CCN3	cellular coı	ENSG000(	0.0182	1	0.349	2.604	1.381	144.8
**52**	CCNDBP1	cyclin D1 k	ENSG000(	0.0273	1	0.394	1.507	0.592	26.849
**53**	CD47	CD47 mole	ENSG000(	0.0286	1	0.401	1.514	0.598	13.966
**54**	CD48	CD48 mole	ENSG000(	0.0138	1	0.323	-2.078	-1.055	16.573
**55**	CD3D	CD3 delta	ENSG000(	0.0359	1	0.428	-1.759	-0.815	22.427
**56**	CD3E	CD3 epsilc	ENSG000(	0.0307	1	0.41	-1.635	-0.709	21.782
**57**	CD79A	CD79a mo	ENSG000(	1.43E-06	0.0387	0.00342	-6.928	-2.792	26.931
**58**	CDKN2AIF	CDKN2A ii	ENSG000(	0.0326	1	0.422	1.621	0.697	14.655
**59**	CDO1	cysteine di	ENSG000(	0.00126	1	0.146	2.839	1.505	23.778
**60**	CDS2	CDP-diacy	ENSG000(	0.0216	1	0.364	1.648	0.721	14.857
**61**	CEMIP	cell migrati	ENSG000(	0.0188	1	0.353	-2.395	-1.26	14.597
**62**	CES1	carboxyles	ENSG000(	0.0326	1	0.422	1.885	0.915	23.517
**63**	CFL2	cofilin 2	ENSG000(	0.00532	1	0.243	2.412	1.27	24.938
**64**	CHI3L2	chitinase 3	ENSG000(	8.33E-05	1	0.045	-8.853	-3.146	21.714
**65**	CHPF	chondroitir	ENSG000(	0.0397	1	0.444	1.611	0.688	24.504
**66**	CIITA	class II ma	ENSG000(	0.00761	1	0.264	-1.867	-0.901	14.9
**67**	CIMAP1B	ciliary micr	ENSG000(	0.00023	1	0.0746	-2.402	-1.264	17.662
**68**	CLEC2D	C-type lect	ENSG000(	0.035	1	0.427	-1.602	-0.68	11.133
**69**	CLEC4E	C-type lect	ENSG000(	0.0176	1	0.344	-1.905	-0.93	11.159
**70**	CLIC4	chloride int	ENSG000(	0.014	1	0.323	1.875	0.907	112.124
**71**	CNN1	calponin 1	ENSG000(	0.017	1	0.342	2.327	1.218	166.748
**72**	COL14A1	collagen ty	ENSG000(	0.00254	1	0.179	3.063	1.615	54.388
**73**	COL8A1	collagen ty	ENSG000(	0.0205	1	0.359	2.261	1.177	39.096
**74**	COPS4	COP9 sign	ENSG000(	0.0214	1	0.364	1.629	0.704	10.349
**75**	COPS8	COP9 sign	ENSG000(	0.0447	1	0.458	1.509	0.593	32.586
**76**	CORO1A	coronin 1A	ENSG000(	0.00381	1	0.214	-1.812	-0.858	23.939
**77**	COX8A	cytochrom	ENSG000(	0.0324	1	0.422	-1.697	-0.763	215.198
**78**	CPED1	cadherin lil	ENSG000(	0.00439	1	0.226	2.189	1.13	12.065
**79**	CPQ	carboxype	ENSG000(	0.047	1	0.464	1.41	0.496	28.274
**80**	CPSF1	cleavage a	ENSG000(	0.00864	1	0.27	-1.773	-0.827	12.632
**81**	CRISPLD1	cysteine ric	ENSG000(	0.00279	1	0.189	2.609	1.384	59.464
**82**	CRTAC1	cartilage a	ENSG000(	0.00665	1	0.258	-1.957	-0.969	38.333
**83**	CRYZ	crystallin z	ENSG000(	0.0332	1	0.423	1.502	0.587	14.435
**84**	CSDC2	cold shock	ENSG000(	0.0136	1	0.321	2.491	1.317	15.515
**85**	CST6	cystatin E/	ENSG000(	0.0294	1	0.404	1.899	0.925	29.957
**86**	CST7	cystatin F	ENSG000(	0.0237	1	0.377	-1.926	-0.945	15.998
**87**	CTC_513N	--	ENSG000(	0.000461	1	0.0988	2.133	1.093	121.63
**88**	CTD_2139	--	ENSG000(	0.0103	1	0.286	-2.305	-1.205	33.169
**89**	CTD_2616	--	ENSG000(	0.021	1	0.361	-1.697	-0.763	97.541
**90**	CTSF	cathepsin I	ENSG000(	0.0205	1	0.359	1.539	0.622	36.337
**91**	CXCL13	C-X-C mot	ENSG000(	2.19E-08	0.000592	0.000157	-11.72	-3.551	13.063
**92**	CXCL16	C-X-C mot	ENSG000(	0.0149	1	0.332	-1.976	-0.983	43.991
**93**	CYBRD1	cytochrom	ENSG000(	0.0108	1	0.288	1.877	0.908	88.988
**94**	CYC1	cytochrom	ENSG000(	0.0216	1	0.364	-1.578	-0.658	21.234
**95**	CYP1B1	cytochrom	ENSG000(	3.77E-05	1	0.0284	-3.868	-1.952	14.696
**96**	CYP51A1	cytochrom	ENSG000(	0.00206	1	0.166	2.127	1.089	20.909
**97**	CYTH1	cytohesin '	ENSG000(	0.0143	1	0.326	-1.718	-0.781	17.596
**98**	CYTH4	cytohesin ⁴	ENSG000(	0.0182	1	0.349	-1.691	-0.758	22.446
**99**	CZIB	CXXC mot	ENSG000(	0.0032	1	0.203	2.676	1.42	15.714
**100**	DAZAP1	DAZ assoc	ENSG000(	0.0157	1	0.335	-1.575	-0.655	14.188
**101**	DEF6	DEF6 guar	ENSG000(	0.0176	1	0.344	-1.789	-0.839	26.576
**102**	DENND1C	DENN don	ENSG000(	0.000599	1	0.108	-3.029	-1.599	15.523

**103**	DHRS4	dehydroge	ENSG000(	0.0174	1	0.343	1.637	0.711	16.051
**104**	DHRSX	dehydroge	ENSG000(	0.0184	1	0.35	-1.596	-0.674	11.484
**105**	DKK3	dickkopf W	ENSG000(	0.0108	1	0.288	2.19	1.131	136.688
**106**	DMPK	DM1 prote	ENSG000(	0.009	1	0.274	2.172	1.119	16.745
**107**	DNAJB4	DnaJ heat	ENSG000(	0.0192	1	0.353	1.891	0.919	29.951
**108**	DOC2B	double C2	ENSG000(	0.00205	1	0.166	-2.305	-1.205	10.362
**109**	DOK2	docking pr	ENSG000(	0.0241	1	0.377	-1.901	-0.927	47.244
**110**	DPT	dermatopo	ENSG000(	0.013	1	0.316	2.031	1.022	22.03
**111**	DSTN	destrin, ac	ENSG000(	0.0095	1	0.28	2.352	1.234	716.575
**112**	DUSP23	dual specif	ENSG000(	0.00661	1	0.258	-1.872	-0.904	103.565
**113**	DYNLT3	dynein ligh	ENSG000(	0.0266	1	0.393	1.557	0.639	39.761
**114**	ECM2	extracellul	ENSG000(	0.000536	1	0.101	3.669	1.875	24.237
**115**	ECRG4	ECRG4 au	ENSG000(	0.0424	1	0.455	1.943	0.958	27.82
**116**	EGFL7	EGF like d	ENSG000(	0.00448	1	0.226	-2.094	-1.066	22.593
**117**	EGLN1	egl-9 famil	ENSG000(	0.0359	1	0.428	1.542	0.625	11.867
**118**	EIF1B	eukaryotic	ENSG000(	0.00549	1	0.244	1.693	0.76	65.411
**119**	ELK3	ETS transc	ENSG000(	0.0294	1	0.404	-1.565	-0.646	38.132
**120**	EMP2	epithelial n	ENSG000(	0.0111	1	0.294	1.91	0.934	14.516
**121**	EPDR1	ependymir	ENSG000(	0.0189	1	0.353	1.854	0.891	43.473
**122**	EPHB4	EPH recep	ENSG000(	0.00661	1	0.258	-1.976	-0.983	11.372
**123**	EPRS1	glutamyl-p	ENSG000(	0.0356	1	0.428	1.499	0.584	16.211
**124**	FABP4	fatty acid b	ENSG000(	0.00443	1	0.226	-3.288	-1.717	63.583
**125**	FADS2	fatty acid c	ENSG000(	0.0454	1	0.459	1.817	0.861	13.762
**126**	FAM157A/	family with	ENSG000(	0.00202	1	0.166	-14.929	-3.9	16.353
**127**	FAM167B	family with	ENSG000(	0.0165	1	0.339	-2.401	-1.263	10.126
**128**	FAM180A	family with	ENSG000(	0.00832	1	0.266	1.978	0.984	16.889
**129**	FAM229B	family with	ENSG000(	0.0345	1	0.426	1.744	0.803	10.663
**130**	FBXL3	F-box and	ENSG000(	0.0306	1	0.41	1.669	0.739	15.028
**131**	FBXO8	F-box prot	ENSG000(	0.0343	1	0.426	1.501	0.585	14.15
**132**	FCN1	ficolin 1	ENSG000(	0.0123	1	0.309	-1.983	-0.987	51.584
**133**	FERMT2	FERM don	ENSG000(	0.014	1	0.323	2.052	1.037	28.466
**134**	FEZ2	fasciculatio	ENSG000(	0.0417	1	0.451	1.467	0.553	10.526
**135**	FGF7	fibroblast g	ENSG000(	0.0158	1	0.335	-2.016	-1.011	12.131
**136**	FGL2	fibrinogen	ENSG000(	0.0464	1	0.463	1.722	0.784	59.527
**137**	FHL5	four and a	ENSG000(	0.0012	1	0.145	4.704	2.234	33.869
**138**	FLNA	filamin A	ENSG000(	0.0104	1	0.286	2.362	1.24	201.634
**139**	FLOT2	flotillin 2	ENSG000(	0.0305	1	0.41	-1.556	-0.638	43.24
**140**	FMO2	flavin cont	ENSG000(	0.000607	1	0.108	2.85	1.511	23.065
**141**	FOXC1	forkhead b	ENSG000(	0.0253	1	0.384	1.963	0.973	23.159
**142**	FOXL1	forkhead b	ENSG000(	0.0174	1	0.343	2.305	1.205	10.333
**143**	FRZB	frizzled rel	ENSG000(	0.00091	1	0.133	3.936	1.977	149.206
**144**	FTH1	ferritin hea	ENSG000(	0.0348	1	0.426	-2.016	-1.011	4090.322
**145**	FTL	ferritin ligh	ENSG000(	0.0281	1	0.399	-2.249	-1.169	32749.03
**146**	FZD1	frizzled cla	ENSG000(	0.022	1	0.367	1.785	0.836	46.2
**147**	G0S2	G0/G1 swi	ENSG000(	0.00492	1	0.232	-3.405	-1.768	130.215
**148**	GET1	guided ent	ENSG000(	0.0367	1	0.431	1.499	0.584	10.86
**149**	GIMAP2	GTPase, II	ENSG000(	0.018	1	0.347	-1.643	-0.716	27.878
**150**	GLS	glutaminas	ENSG000(	0.0272	1	0.394	1.779	0.831	42.316
**151**	GMIP	GEM intera	ENSG000(	0.0269	1	0.394	-1.738	-0.798	14.887
**152**	GMPR2	guanosine	ENSG000(	0.00312	1	0.202	1.771	0.825	10.51
**153**	GNA15	G protein s	ENSG000(	0.00792	1	0.265	-1.833	-0.874	12.643
**154**	GNG12	G protein s	ENSG000(	0.0232	1	0.374	1.805	0.852	64.901

**155**	GNPNAT1	glucosami	ENSG000(	0.0166	1	0.34	1.583	0.663	12.578
**156**	GOLIM4	golgi integ	ENSG000(	0.0241	1	0.377	1.669	0.739	26.103
**157**	GPR174	G protein-c	ENSG000(	0.0325	1	0.422	-1.847	-0.885	15.601
**158**	GPSM3	G protein s	ENSG000(	0.00156	1	0.159	-2.157	-1.109	36.714
**159**	GTF2H5	general tra	ENSG000(	0.00909	1	0.276	1.631	0.706	10.166
**160**	GUCY1A1	guanylate	ENSG000(	0.0359	1	0.428	1.682	0.75	22.256
**161**	GUCY1B1	guanylate	ENSG000(	0.0425	1	0.455	1.752	0.809	10.313
**162**	GULP1	GULP PTE	ENSG000(	0.00607	1	0.256	2.616	1.387	11.205
**163**	H1-1	H1.1 linker	ENSG000(	0.0204	1	0.358	2.198	1.136	10.033
**164**	H2AC20	H2A cluste	ENSG000(	0.0355	1	0.428	-1.606	-0.684	224.759
**165**	HCFC1R1	host cell fa	ENSG000(	0.00776	1	0.265	1.826	0.869	63.478
**166**	HCST	hematopoi	ENSG000(	0.0243	1	0.377	-1.714	-0.777	97.407
**167**	HDAC3	histone de	ENSG000(	0.0341	1	0.426	-1.567	-0.648	12.493
**168**	HDAC7	histone de	ENSG000(	0.0316	1	0.418	-1.5	-0.585	18.292
**169**	HES4	hes family	ENSG000(	0.00229	1	0.172	2.211	1.145	17.788
**170**	HIGD1A	HIG1 hypo	ENSG000(	0.0246	1	0.379	1.605	0.683	12.34
**171**	HK3	hexokinase	ENSG000(	0.0393	1	0.443	-1.807	-0.854	16.695
**172**	HMCN1	hemicentin	ENSG000(	0.00938	1	0.278	2.523	1.335	10.481
**173**	HNRNPF	heterogene	ENSG000(	0.0325	1	0.422	-1.566	-0.647	12.562
**174**	HNRNPH3	heterogene	ENSG000(	0.018	1	0.347	1.553	0.635	17.703
**175**	HSPA6	heat shock	ENSG000(	0.0197	1	0.356	-1.853	-0.89	26.835
**176**	HSPB6	heat shock	ENSG000(	0.0454	1	0.459	1.775	0.828	90.159
**177**	HSPB7	heat shock	ENSG000(	0.0052	1	0.24	3.415	1.772	50.187
**178**	HYAL2	hyaluronid	ENSG000(	0.0044	1	0.226	-1.775	-0.828	11.084
**179**	ID3	inhibitor of	ENSG000(	1.07E-05	0.29	0.0135	6.297	2.655	23.339
**180**	IER3	immediate	ENSG000(	0.0036	1	0.212	-2.19	-1.131	104.755
**181**	IFITM3	interferon i	ENSG000(	0.0355	1	0.428	-1.573	-0.653	163.06
**182**	IGFBP3	insulin like	ENSG000(	0.0233	1	0.374	1.995	0.996	70.05
**183**	IGFBP4	insulin like	ENSG000(	0.0341	1	0.426	-1.947	-0.961	144.568
**184**	IGFBP6	insulin like	ENSG000(	0.0333	1	0.423	1.899	0.925	515.572
**185**	IGFBP7	insulin like	ENSG000(	0.047	1	0.464	1.753	0.809	725.815
**186**	IGLL1/IGLl	immunoglo	ENSG000(	0.0249	1	0.381	-3.11	-1.637	208.534
**187**	IL6	interleukin	ENSG000(	6.09E-05	1	0.0365	-7.686	-2.942	56.014
**188**	IL15RA	interleukin	ENSG000(	0.0436	1	0.458	-1.546	-0.628	14.281
**189**	IL1RN	interleukin	ENSG000(	0.0084	1	0.266	-2.509	-1.327	10.634
**190**	IL2RG	interleukin	ENSG000(	0.0284	1	0.4	-1.687	-0.754	15.983
**191**	IL4R	interleukin	ENSG000(	0.0373	1	0.434	-1.679	-0.747	16.193
**192**	INPP5A	inositol pol	ENSG000(	0.0415	1	0.451	1.757	0.813	22.986
**193**	IRAG1	inositol 1,4	ENSG000(	0.0164	1	0.339	2.186	1.128	16.79
**194**	ITFG1	integrin alp	ENSG000(	0.00948	1	0.28	1.664	0.734	16.585
**195**	ITGA1	integrin su	ENSG000(	0.0202	1	0.358	2.144	1.1	28.924
**196**	ITGA3	integrin su	ENSG000(	0.00888	1	0.273	1.749	0.806	17.113
**197**	ITGA8	integrin su	ENSG000(	0.0243	1	0.377	2.467	1.303	110.335
**198**	ITGB5	integrin su	ENSG000(	0.0254	1	0.384	1.764	0.819	22.936
**199**	IWS1	interacts w	ENSG000(	0.0168	1	0.341	1.647	0.72	10.836
**200**	JCHAIN	joining cha	ENSG000(	0.00348	1	0.212	-3.096	-1.63	114.977
**201**	KANK1	KN motif a	ENSG000(	0.0223	1	0.37	2.018	1.013	24.417
**202**	KANK2	KN motif a	ENSG000(	0.0367	1	0.431	1.683	0.751	16.968
**203**	KCNAB2	potassium	ENSG000(	0.0339	1	0.426	-1.779	-0.831	10.318
**204**	KCNN4	potassium	ENSG000(	0.0438	1	0.458	-1.722	-0.784	17.353
**205**	KCTD5	potassium	ENSG000(	0.0493	1	0.469	-1.545	-0.627	21.681
**206**	KCTD9	potassium	ENSG000(	0.000783	1	0.121	2.159	1.11	15.794

**207**	KIDINS22(	kinase D ir	ENSG000(	0.0327	1	0.422	1.568	0.649	10.06
**208**	KLHDC2	kelch dom:	ENSG000(	0.0479	1	0.465	1.475	0.561	27.538
**209**	KPNA2P3	--	ENSG000(	0.0058	1	0.252	-1.729	-0.79	144.494
**210**	LAMB2	laminin sul	ENSG000(	0.0377	1	0.435	1.518	0.603	14.858
**211**	LAMC1	laminin sul	ENSG000(	0.00252	1	0.179	1.893	0.921	18.469
**212**	LCE3E	late cornifi	ENSG000(	0.000312	1	0.0801	-5.904	-2.562	12.035
**213**	LDAF1	lipid drople	ENSG000(	0.0459	1	0.46	1.594	0.672	12.25
**214**	LDOC1	LDOC1 re(	ENSG000(	0.000531	1	0.101	3.465	1.793	148.396
**215**	LFNG	LFNG O-fu	ENSG000(	0.00173	1	0.164	-2.151	-1.105	13.772
**216**	LGALS2	galectin 2	ENSG000(	0.0175	1	0.344	-3.235	-1.694	12.114
**217**	LGR6	leucine ricl	ENSG000(	0.00151	1	0.159	3.179	1.669	14.101
**218**	LILRB2	leukocyte i	ENSG000(	0.0131	1	0.317	-1.86	-0.896	21.418
**219**	LIMD2	LIM domai	ENSG000(	0.00537	1	0.243	-1.698	-0.764	13.282
**220**	LPAR1	lysophospl	ENSG000(	0.00735	1	0.264	2.02	1.014	20.177
**221**	LRRC57	leucine ricl	ENSG000(	0.0445	1	0.458	1.469	0.555	10.844
**222**	LTB	lymphotoxi	ENSG000(	1.07E-06	0.0291	0.00308	-4.288	-2.1	21.674
**223**	LTBP1	latent trans	ENSG000(	0.0257	1	0.386	2.351	1.233	11.954
**224**	MAGEH1	MAGE fam	ENSG000(	0.0141	1	0.324	1.747	0.805	32.179
**225**	MAGT1	magnesiur	ENSG000(	0.0209	1	0.36	1.702	0.767	15.79
**226**	MAOA	monoamin	ENSG000(	0.00726	1	0.264	2.146	1.101	11.505
**227**	MAP1LC3/	microtubul	ENSG000(	0.0197	1	0.356	1.695	0.761	33.539
**228**	MAP3K11	mitogen-ac	ENSG000(	0.0467	1	0.464	-1.585	-0.664	14.257
**229**	MARCKSL	MARCKS l	ENSG000(	0.0225	1	0.37	-2.17	-1.118	12.683
**230**	MCAM	melanoma	ENSG000(	0.0333	1	0.423	2.02	1.014	151.699
**231**	MED18	mediator c	ENSG000(	0.0449	1	0.459	1.495	0.58	12.573
**232**	METTL5	methyltran	ENSG000(	0.0173	1	0.343	1.622	0.698	25.86
**233**	MFAP4	microfibril :	ENSG000(	0.00402	1	0.218	3.256	1.703	525.436
**234**	MFGE8	milk fat glo	ENSG000(	0.0181	1	0.348	2.033	1.024	255.398
**235**	MGST3	microsoma	ENSG000(	0.0128	1	0.314	1.815	0.86	37.844
**236**	MIB1	MIB E3 ub	ENSG000(	0.0187	1	0.353	1.699	0.765	11.471
**237**	MOB4	MOB famil	ENSG000(	0.0161	1	0.338	1.665	0.736	22.445
**238**	MPZL2	myelin pro	ENSG000(	0.0285	1	0.4	-1.789	-0.839	10.932
**239**	MRAP2	melanocor	ENSG000(	0.00399	1	0.218	2.956	1.564	50.586
**240**	MRGPRF	MAS relate	ENSG000(	0.0316	1	0.418	1.806	0.853	12.332
**241**	MRPL12	mitochondi	ENSG000(	0.0168	1	0.342	-1.736	-0.796	38.537
**242**	MRPS18B	mitochondi	ENSG000(	0.023	1	0.373	1.501	0.586	26.829
**243**	MS4A7	membrane	ENSG000(	0.0272	1	0.394	-1.88	-0.91	18.109
**244**	MSMO1	methylster	ENSG000(	0.00439	1	0.226	2.176	1.122	13.359
**245**	MT1X	metallothic	ENSG000(	0.0215	1	0.364	-2.127	-1.089	48.311
**246**	MT2A	metallothic	ENSG000(	0.00552	1	0.245	-3.078	-1.622	538.911
**247**	MTARC2	mitochondi	ENSG000(	0.0264	1	0.391	1.723	0.785	11.06
**248**	MXI1	MAX intera	ENSG000(	0.0261	1	0.388	1.804	0.851	31.519
**249**	MYDGF	myeloid de	ENSG000(	0.0349	1	0.426	-1.557	-0.638	53.635
**250**	MYH10	myosin hea	ENSG000(	0.0163	1	0.339	2.719	1.443	74.671
**251**	MYL9	myosin ligl	ENSG000(	0.00632	1	0.258	2.442	1.288	2033.948
**252**	MYO1D	myosin ID	ENSG000(	0.017	1	0.342	1.912	0.935	53.284
**253**	MYO1F	myosin IF	ENSG000(	0.0197	1	0.356	-1.766	-0.821	20.508
**254**	MYOF	myoferlin	ENSG000(	0.0473	1	0.465	1.469	0.554	15.38
**255**	MYOZ1	myozenin	ENSG000(	0.0021	1	0.167	3.027	1.598	12.837
**256**	MZB1	marginal z	ENSG000(	0.000045	1	0.0294	-4.708	-2.235	16.393
**257**	NAA10	N-alpha-ac	ENSG000(	0.0169	1	0.342	-1.721	-0.783	23.051
**258**	NAPSB	napsin B a	ENSG000(	0.00144	1	0.158	-3.085	-1.625	14.484

**259**	NCK1	NCK adap	ENSG000(	0.0407	1	0.447	1.43	0.516	13.273
**260**	NCOR2	nuclear re	ENSG000(	0.0258	1	0.386	-1.556	-0.638	11.847
**261**	NDUFA4	NDUFA4 n	ENSG000(	0.011	1	0.291	1.837	0.877	70.168
**262**	NDUFAF2	NADH:ubic	ENSG000(	0.0468	1	0.464	1.527	0.611	64.672
**263**	NDUFB4	NADH:ubic	ENSG000(	0.0356	1	0.428	1.53	0.613	54.156
**264**	NEK3	NIMA relat	ENSG000(	0.0356	1	0.428	-1.525	-0.609	10.137
**265**	NEO1	neogenin 1	ENSG000(	0.0387	1	0.44	1.754	0.811	11.335
**266**	NEURL2	neuralized	ENSG000(	0.0239	1	0.377	-1.927	-0.947	11.074
**267**	NHERF1	NHERF fa	ENSG000(	0.0233	1	0.374	-1.699	-0.765	15.898
**268**	NPEPL1	aminopept	ENSG000(	0.0328	1	0.422	-1.499	-0.584	11.734
**269**	NPNT	nephronec	ENSG000(	0.00132	1	0.149	3.414	1.771	12.168
**270**	NQO1	NAD(P)H (	ENSG000(	0.00723	1	0.264	2.01	1.007	10.151
**271**	NTN4	netrin 4	ENSG000(	0.031	1	0.412	1.778	0.83	36.768
**272**	NXPH3	neurexoph	ENSG000(	0.0466	1	0.463	1.793	0.843	20.629
**273**	OAT	ornithine a	ENSG000(	0.00645	1	0.258	1.755	0.812	38.54
**274**	OAZ1	ornithine d	ENSG000(	0.049	1	0.468	-1.588	-0.668	89.351
**275**	OGN	osteoglycir	ENSG000(	0.00463	1	0.229	3.005	1.587	150.253
**276**	OLFML1	olfactomec	ENSG000(	0.00288	1	0.192	2.095	1.067	14.624
**277**	OMD	osteomodu	ENSG000(	0.0113	1	0.296	2.577	1.366	50.709
**278**	OXR1	oxidation r	ENSG000(	0.00857	1	0.269	1.884	0.914	11.856
**279**	PAM	peptidylgly	ENSG000(	0.00356	1	0.212	2.275	1.186	20.887
**280**	PARM1	prostate ar	ENSG000(	0.00442	1	0.226	2.605	1.381	28.221
**281**	PARP14	poly(ADP-r	ENSG000(	0.0395	1	0.444	-1.498	-0.583	11.11
**282**	PCED1A	PC-esteras	ENSG000(	0.00113	1	0.141	-2.288	-1.194	13.794
**283**	PCYOX1	prenylcyste	ENSG000(	0.0144	1	0.327	1.699	0.765	11.015
**284**	PDE3A	phosphodi	ENSG000(	0.0239	1	0.377	1.79	0.84	19.22
**285**	PF4V1	platelet fac	ENSG000(	0.00408	1	0.22	-4.904	-2.294	24.586
**286**	PFN2	profilin 2	ENSG000(	0.00155	1	0.159	2.964	1.568	43.244
**287**	PGGHG	protein-glu	ENSG000(	0.00829	1	0.266	-1.733	-0.794	11.2
**288**	PGM5	phosphogl	ENSG000(	0.0146	1	0.328	2.124	1.087	15.988
**289**	PGRMC2	progestero	ENSG000(	0.0139	1	0.323	1.749	0.806	15.793
**290**	PGS1	phosphatic	ENSG000(	0.0089	1	0.273	-1.795	-0.844	10.787
**291**	PHAX	phosphory	ENSG000(	0.0349	1	0.426	1.523	0.607	12.155
**292**	PHF10	PHD finger	ENSG000(	0.0174	1	0.343	1.657	0.728	27.791
**293**	PIGH	phosphatic	ENSG000(	0.0268	1	0.394	1.551	0.633	10.867
**294**	PIM2	Pim-2 prot	ENSG000(	0.000343	1	0.0853	-3.179	-1.669	27.997
**295**	PKD2	polycystin	ENSG000(	0.026	1	0.387	1.896	0.923	107.632
**296**	PLEKHA4	pleckstrin l	ENSG000(	0.0177	1	0.344	-1.589	-0.669	19.077
**297**	PLEKHM1	--	ENSG000(	0.0105	1	0.287	-1.712	-0.776	20.719
**298**	PLS3	plastin 3	ENSG000(	0.00172	1	0.164	2.908	1.54	236.541
**299**	PLSCR4	phospholip	ENSG000(	0.00185	1	0.166	2.549	1.35	44.758
**300**	PLVAP	plasmalem	ENSG000(	0.0114	1	0.296	-2.075	-1.053	92.724
**301**	PNMA8A	PNMA fam	ENSG000(	0.000439	1	0.0978	3.164	1.662	11.571
**302**	POLD4	DNA polyn	ENSG000(	0.0445	1	0.458	-1.514	-0.598	46.846
**303**	POMZP3	POM121 a	ENSG000(	0.00658	1	0.258	2.072	1.051	15.797
**304**	PPAN	peter pan l	ENSG000(	0.019	1	0.353	-1.594	-0.673	14.549
**305**	PPP2CB	protein pho	ENSG000(	0.0158	1	0.335	1.573	0.654	33.209
**306**	PPP6R1	protein pho	ENSG000(	0.0209	1	0.36	-1.683	-0.751	20.816
**307**	PRDM6	PR/SET do	ENSG000(	0.0244	1	0.377	1.967	0.976	10.248
**308**	PRKG1	protein kin	ENSG000(	0.0145	1	0.328	2.377	1.249	22.37
**309**	PROCR	protein C r	ENSG000(	0.047	1	0.464	-1.603	-0.681	27.245
**310**	PROS1	protein S	ENSG000(	0.00181	1	0.166	2.387	1.255	34.525

**311**	PRR13	proline rich	ENSG0000(	0.00336	1	0.208	-1.809	-0.855	29.837
**312**	PSMC5	proteasom	ENSG0000(	0.048	1	0.465	-1.485	-0.57	28.127
**313**	PSMD4	proteasom	ENSG0000(	0.037	1	0.431	-1.571	-0.652	43.591
**314**	PSME2	proteasom	ENSG0000(	0.0418	1	0.451	-1.556	-0.638	12.741
**315**	PTGDS	prostaglan	ENSG0000(	0.0076	1	0.264	-2.485	-1.313	18.586
**316**	PTK2B	protein tyr(	ENSG0000(	0.00183	1	0.166	-2.024	-1.018	14.156
**317**	PTPN18	protein tyr(	ENSG0000(	0.0463	1	0.462	-1.494	-0.579	12.658
**318**	PXDC1	PX domair	ENSG0000(	0.0269	1	0.394	1.689	0.756	77.471
**319**	PYGB	glycogen p	ENSG0000(	0.03	1	0.408	1.587	0.666	37.874
**320**	R3HDM4	R3H doma	ENSG0000(	0.0163	1	0.339	-1.595	-0.673	32.4
**321**	RAB14	RAB14, m	ENSG0000(	0.0161	1	0.338	1.568	0.649	22.167
**322**	RAB22A	RAB22A, r	ENSG0000(	0.0487	1	0.468	1.491	0.576	13.52
**323**	RAB2A	RAB2A, m	ENSG0000(	0.0383	1	0.439	1.586	0.666	23.677
**324**	RAB3IL1	RAB3A int	ENSG0000(	0.0385	1	0.439	-1.708	-0.772	21.345
**325**	RABAC1	Rab accep	ENSG0000(	0.0177	1	0.344	1.668	0.738	41.196
**326**	RABGAP1	RAB GTPa	ENSG0000(	0.023	1	0.373	1.731	0.792	12.761
**327**	RALGDS	ral guanine	ENSG0000(	0.0419	1	0.451	-1.501	-0.586	10.33
**328**	RASIP1	Ras intera	ENSG0000(	0.00368	1	0.212	-2.222	-1.152	17.687
**329**	RASL12	RAS like fa	ENSG0000(	0.0399	1	0.444	1.986	0.99	25.116
**330**	RASL11B	RAS like fa	ENSG0000(	0.00459	1	0.227	2.783	1.477	11.104
**331**	RBMS3	RNA bindi	ENSG0000(	0.0192	1	0.353	1.892	0.92	14.51
**332**	RCN2	reticulocall	ENSG0000(	0.0439	1	0.458	1.528	0.612	16.022
**333**	RERG	RAS like e	ENSG0000(	0.013	1	0.316	2.163	1.113	13.497
**334**	RERGL	RERG like	ENSG0000(	0.000136	1	0.0613	6.406	2.679	19.161
**335**	RGS5	regulator o	ENSG0000(	0.0459	1	0.46	2.097	1.068	292.714
**336**	RGS16	regulator o	ENSG0000(	3.47E-05	0.941	0.0277	-3.062	-1.614	12.767
**337**	RNF20	ring finger	ENSG0000(	0.0179	1	0.347	1.548	0.63	16.272
**338**	RNF149	ring finger	ENSG0000(	0.0417	1	0.451	-1.541	-0.624	18.647
**339**	RNF180	ring finger	ENSG0000(	0.0382	1	0.438	1.718	0.78	12.034
**340**	ROCK2	Rho assoc	ENSG0000(	0.00314	1	0.203	1.957	0.969	16.424
**341**	RP11_34P	--	ENSG0000(	0.00203	1	0.166	-2.617	-1.388	90.469
**342**	RP4_583P	--	ENSG0000(	0.00362	1	0.212	-2.438	-1.286	17.513
**343**	RPA2	replication	ENSG0000(	0.0351	1	0.428	-1.497	-0.582	26.591
**344**	RPE	ribulose-5-	ENSG0000(	0.0154	1	0.335	1.616	0.692	28.638
**345**	RPL39L	ribosomal	ENSG0000(	0.0148	1	0.33	1.89	0.918	14.259
**346**	RPS29	ribosomal	ENSG0000(	0.0385	1	0.439	1.63	0.705	12.552
**347**	RPS6KA2	ribosomal	ENSG0000(	0.0328	1	0.422	-1.599	-0.678	13.117
**348**	RRAD	RRAD, Ra	ENSG0000(	0.0077	1	0.264	2.186	1.128	18.92
**349**	RRAGA	Ras relate	ENSG0000(	0.0425	1	0.455	1.471	0.557	49.943
**350**	RRN3P2	RRN3 pse	ENSG0000(	0.0437	1	0.458	-1.672	-0.742	34.707
**351**	RSL24D1	ribosomal	ENSG0000(	0.0372	1	0.433	1.544	0.626	36.389
**352**	RTCA	RNA 3'-ter	ENSG0000(	0.011	1	0.291	-1.766	-0.82	39.974
**353**	RXRA	retinoid X r	ENSG0000(	0.0188	1	0.353	-1.667	-0.737	12.23
**354**	S100A8	S100 calci	ENSG0000(	0.0421	1	0.453	-1.796	-0.845	85.603
**355**	S100A9	S100 calci	ENSG0000(	0.0165	1	0.339	-1.92	-0.941	464.932
**356**	S1PR1	sphingosin	ENSG0000(	0.0303	1	0.41	-1.736	-0.796	14.214
**357**	SAMD3	sterile alph	ENSG0000(	0.0341	1	0.426	-1.579	-0.659	11.554
**358**	SBNO2	strawberry	ENSG0000(	0.000655	1	0.114	-1.957	-0.968	16.019
**359**	SCARA3	scavenger	ENSG0000(	0.00163	1	0.161	2.454	1.295	20.299
**360**	SDSL	serine deh	ENSG0000(	0.0214	1	0.364	-1.864	-0.899	12.278
**361**	SEC23A	SEC23 ho	ENSG0000(	0.0276	1	0.394	1.562	0.643	10.71
**362**	SEC31A	SEC31 ho	ENSG0000(	0.0397	1	0.444	1.545	0.628	22.09

**363**	SEPTIN6	septin 6	ENSG000(	0.0391	1	0.442	-1.477	-0.563	15.241
**364**	SEPTIN10	septin 10	ENSG000(	0.0215	1	0.364	1.668	0.738	25.268
**365**	SERINC3	serine incc	ENSG000(	0.0133	1	0.318	1.643	0.717	32.098
**366**	SFRP2	secreted fr	ENSG000(	0.00397	1	0.218	-2.236	-1.161	95.107
**367**	SGCB	sarcoglyca	ENSG000(	0.000994	1	0.136	2.452	1.294	23.986
**368**	SGSM3	small G pr	ENSG000(	0.0067	1	0.258	-1.855	-0.891	14.629
**369**	SH3BGR	SH3 doma	ENSG000(	0.000918	1	0.133	3.733	1.9	26.73
**370**	SHC1	SHC adap	ENSG000(	0.0348	1	0.426	-1.531	-0.614	62.29
**371**	SLAIN2	SLAIN mot	ENSG000(	0.0123	1	0.309	1.787	0.838	23.545
**372**	SLC22A3	solute carr	ENSG000(	0.002	1	0.166	2.458	1.298	18.022
**373**	SLC25A4	solute carr	ENSG000(	0.00247	1	0.179	2.473	1.306	20.5
**374**	SLC2A1	solute carr	ENSG000(	0.049	1	0.468	1.771	0.824	22.343
**375**	SLCO2A1	solute carr	ENSG000(	0.000255	1	0.0765	-2.417	-1.273	21.978
**376**	SMOC2	SPARC rel	ENSG000(	0.00678	1	0.258	2.6	1.378	118.297
**377**	SNHG8	small nucle	ENSG000(	0.0288	1	0.401	-1.928	-0.947	575.565
**378**	SNHG12	small nucle	ENSG000(	0.00562	1	0.248	-3.253	-1.702	1570.665
**379**	SOCS3	suppresso	ENSG000(	4.8E-07	0.013	0.00172	-3.964	-1.987	39.011
**380**	SOD1	superoxide	ENSG000(	0.0224	1	0.37	1.848	0.886	314.872
**381**	SORT1	sortilin 1	ENSG000(	0.00835	1	0.266	1.969	0.977	36.093
**382**	SOX18	SRY-box tr	ENSG000(	0.00491	1	0.232	-2.534	-1.341	20.27
**383**	SPCS2	signal pept	ENSG000(	0.0471	1	0.464	1.499	0.584	69.145
**384**	SPHK1	sphingosin	ENSG000(	0.00357	1	0.212	-2.117	-1.082	12.532
**385**	SPRYD3	SPRY dom	ENSG000(	0.0444	1	0.458	1.475	0.561	53.912
**386**	SRSF2	serine and	ENSG000(	0.0271	1	0.394	-1.71	-0.774	33.027
**387**	SSB	small RNA	ENSG000(	0.0258	1	0.387	1.565	0.647	14.841
**388**	SSNA1	SS nuclear	ENSG000(	0.0299	1	0.407	-1.565	-0.646	73.551
**389**	ST6GAL2	ST6 beta-g	ENSG000(	0.000931	1	0.134	3.576	1.838	13.357
**390**	ST6GALN/	ST6 N-ace	ENSG000(	0.0107	1	0.288	-1.695	-0.761	10.765
**391**	STAB1	stabilin 1	ENSG000(	0.00486	1	0.232	-1.812	-0.858	15.825
**392**	STEEP1	STING1 EI	ENSG000(	0.0428	1	0.457	1.476	0.562	16.126
**393**	STK38L	serine/thre	ENSG000(	0.0435	1	0.458	1.773	0.826	34.433
**394**	STX10	syntaxin 1(	ENSG000(	0.029	1	0.401	-1.465	-0.551	29.066
**395**	STXBP2	syntaxin bi	ENSG000(	0.0318	1	0.419	-1.686	-0.753	18.206
**396**	STXBP3	syntaxin bi	ENSG000(	0.0243	1	0.377	1.633	0.708	15.875
**397**	SUN1	Sad1 and I	ENSG000(	0.00818	1	0.266	1.861	0.896	12.71
**398**	SUSD5	sushi doma	ENSG000(	0.00068	1	0.115	4.103	2.037	55.467
**399**	SVIL	supervillin	ENSG000(	0.0355	1	0.428	1.848	0.886	25.584
**400**	SVIL-AS1	SVIL antis	ENSG000(	0.00115	1	0.141	1.851	0.888	10.802
**401**	SYNPO2	synaptopo	ENSG000(	0.00774	1	0.265	2.493	1.318	22.062
**402**	SYS1-DBN	SYS1-DBN	ENSG000(	0.0154	1	0.335	1.679	0.747	150.041
**403**	SYT11	synaptotag	ENSG000(	0.00252	1	0.179	1.793	0.842	25.205
**404**	TBXA2R	thromboxa	ENSG000(	0.0412	1	0.449	1.796	0.845	16.716
**405**	TCEAL2	transcriptic	ENSG000(	0.0195	1	0.355	2.274	1.185	37.656
**406**	TCIM	transcriptic	ENSG000(	0.00761	1	0.264	-1.901	-0.927	23.866
**407**	TCIRG1	T cell immu	ENSG000(	0.00324	1	0.204	-1.966	-0.976	13.743
**408**	TENT5A	terminal nu	ENSG000(	0.0471	1	0.464	1.482	0.568	13.154
**409**	TES	testin LIM	ENSG000(	0.0282	1	0.399	1.629	0.704	25.901
**410**	TFE3	transcriptic	ENSG000(	0.0489	1	0.468	-1.562	-0.644	18.018
**411**	TINAGL1	tubulointer	ENSG000(	0.0295	1	0.404	1.904	0.929	55.893
**412**	TM2D1	TM2 doma	ENSG000(	0.0325	1	0.422	1.538	0.621	18.4
**413**	TMCO3	transmemb	ENSG000(	0.0427	1	0.456	1.697	0.763	15.991
**414**	TMEM47	transmemb	ENSG000(	0.000961	1	0.136	3.373	1.754	73.36

**415**	TMEM106A	transmemb	ENSG0000(	0.0222	1	0.369	-1.669	-0.739	13.69
**416**	TMEM106(	transmemb	ENSG0000(	0.00102	1	0.136	2.487	1.314	24.439
**417**	TMEM30A	transmemb	ENSG0000(	0.0478	1	0.465	1.596	0.675	49.586
**418**	TMEM30B	transmemb	ENSG0000(	0.0333	1	0.423	1.849	0.887	13.304
**419**	TNFAIP2	TNF alpha	ENSG0000(	0.00318	1	0.203	-2.059	-1.042	14.516
**420**	TNFSF10	TNF super	ENSG0000(	0.0114	1	0.296	-1.704	-0.769	15.46
**421**	TOM1	target of m	ENSG0000(	0.00712	1	0.264	-1.709	-0.773	13.473
**422**	TOP2B	DNA topoi	ENSG0000(	0.0257	1	0.386	1.597	0.675	14.057
**423**	TPM1	tropomyos	ENSG0000(	0.0379	1	0.437	1.927	0.946	90.593
**424**	TRABD	TraB doma	ENSG0000(	0.0016	1	0.16	-1.873	-0.905	29.424
**425**	TRAF3	TNF recep	ENSG0000(	0.0191	1	0.353	-1.669	-0.739	11.634
**426**	TRIB1	tribbles ps	ENSG0000(	0.0184	1	0.35	-1.674	-0.743	10.871
**427**	TRIM8	tripartite m	ENSG0000(	0.0478	1	0.465	-1.629	-0.704	130.016
**428**	TRMT1	tRNA meth	ENSG0000(	0.01	1	0.284	-1.976	-0.983	23.74
**429**	TRPC4AP	transient re	ENSG0000(	0.0493	1	0.469	-1.541	-0.624	51.499
**430**	TRPM2	transient re	ENSG0000(	0.0365	1	0.43	-1.762	-0.817	12.014
**431**	TSPAN2	tetraspanir	ENSG0000(	0.00654	1	0.258	2.149	1.104	23.286
**432**	TSPAN33	tetraspanir	ENSG0000(	0.00656	1	0.258	-1.956	-0.968	11.283
**433**	TSPO	translocato	ENSG0000(	0.0386	1	0.439	-1.599	-0.678	76.929
**434**	TTC1	tetratricope	ENSG0000(	0.0241	1	0.377	1.549	0.632	53.989
**435**	TUBE1	tubulin eps	ENSG0000(	0.0199	1	0.356	1.736	0.796	10.512
**436**	TWSG1	twisted ga	ENSG0000(	0.00205	1	0.166	2.182	1.126	66.162
**437**	TYMP	thymidine		ENSG0000(	0.0442	1	0.458	-1.862	-0.897	122.629
**438**	UBE2D1	ubiquitin c	ENSG0000(	0.0153	1	0.335	1.687	0.754	17.918
**439**	UBE2E2	ubiquitin c	ENSG0000(	0.0254	1	0.384	1.562	0.644	13.262
**440**	UBE2H	ubiquitin c	ENSG0000(	0.00111	1	0.141	2.316	1.211	27.298
**441**	UBE2V2	ubiquitin c	ENSG0000(	0.0208	1	0.36	1.592	0.671	14.424
**442**	UBQLN2	ubiquilin 2	ENSG0000(	0.0193	1	0.353	1.578	0.658	32.058
**443**	UNC50	unc-50 inn	ENSG0000(	0.0302	1	0.41	1.573	0.653	29.557
**444**	UQCRQ	ubiquinol-c	ENSG0000(	0.0498	1	0.47	1.488	0.573	115.93
**445**	VCL	vinculin	ENSG0000(	0.00542	1	0.243	3.134	1.648	10.264
**446**	VDAC3	voltage de	ENSG0000(	0.0498	1	0.47	1.469	0.555	19.487
**447**	VGLL4	vestigial lik	ENSG0000(	0.0335	1	0.425	-1.555	-0.637	12.861
**448**	VPS37C	VPS37C s	ENSG0000(	0.0345	1	0.426	-1.732	-0.793	12.011
**449**	VSIR	V-set immu	ENSG0000(	0.0269	1	0.394	-1.547	-0.629	19.127
**450**	VWF	von Willeb	ENSG0000(	0.023	1	0.373	-2.158	-1.11	11.457
**451**	WAS	WASP act	ENSG0000(	0.0256	1	0.385	-1.746	-0.804	37.165
**452**	WASL	WASP like	ENSG0000(	0.0344	1	0.426	1.561	0.642	28.805
**453**	WTIP	WT1 intera	ENSG0000(	0.00802	1	0.265	2.31	1.208	27.092
**454**	YAP1	Yes1 asso	ENSG0000(	0.0149	1	0.331	2.052	1.037	67.01
**455**	YJU2	YJU2 splic	ENSG0000(	0.00496	1	0.232	-2.057	-1.04	24.543
**456**	YWHAQ	tyrosine 3-	ENSG0000(	0.0227	1	0.372	1.691	0.758	107.735
**457**	ZC3H13	zinc finger	ENSG0000(	0.0108	1	0.288	1.68	0.749	13.875
**458**	ZFAND1	zinc finger	ENSG0000(	0.0359	1	0.428	1.674	0.743	13.411
**459**	ZFP91-CN	ZFP91-CN	ENSG0000(	0.046	1	0.461	1.476	0.561	52.905
**460**	ZMIZ1	zinc finger	ENSG0000(	0.00591	1	0.253	-1.854	-0.891	15.355
**461**	ZNF208	zinc finger	ENSG0000(	0.0386	1	0.439	1.921	0.942	58.446
**462**	ZNF581	zinc finger	ENSG0000(	0.0306	1	0.41	-1.629	-0.704	15.621
**463**	ZNF667-A:	ZNF667 ar	ENSG0000(	0.00724	1	0.264	1.907	0.931	12.746

Location	Type(s)	Biomarker	Drug(s)	Entrez Ger	Entrez Ger	Entrez Gene ID for Rat
Extracellul	transporter			2	232345	24153
Cytoplasm	enzyme			340485	230379	313339
Cytoplasm	other	efficacy		59	11475	81633
Cytoplasm	enzyme			70	11464	29275
Cytoplasm	transcription regulator			81	60595	63836
Cytoplasm	enzyme		N6022	128	11532	1E+08
Cytoplasm	enzyme		fomepizole	125		
Nucleus	enzyme			55256	104923	298934
Cytoplasm	other			64400	14339	291906
Cytoplasm	enzyme	diagnosis,e	balsalazide	240	11689	25290
Extracellul	other			51129	57875	362850
Cytoplasm	other			353322	654824	361149
Plasma Me	ion channel		crofelemer	55107	101772	309135
Plasma Me	ion channel			196527	105722	315272
Plasma Me	transmemb	diagnosis		84168	69538	362393
Plasma Me	ion channel			310	11750	155423
Plasma Me	enzyme		ASP8232,I	8639	11754	29473
Cytoplasm	transporter			1176	11777	302290
Cytoplasm	other			8539	11800	362170
Cytoplasm	enzyme			378	11843	79120
Cytoplasm	other			393	171207	246249
Cytoplasm	other			55156	74252	294948
Other	other			51309	78248	
Cytoplasm	other			81873	74192	296710
Extracellul	other			54829	66695	306805
Plasma Me	enzyme			84896	67979	309532
Cytoplasm	other			25923	109168	309187
Cytoplasm	enzyme			148789	97884	291212
Nucleus	transcription regulator			56987	70508	303970
Cytoplasm	other			118426	67774	362452
Nucleus	transcription regulator			25855	107392	
Nucleus	other			84419	433470	302884
Cytoplasm	other			29071	59048	302499
Other	other			127687		362592
Cytoplasm	other			84886	69551	498967
Extracellul	other			114898	69183	497886
Other	other			1E+08	1E+08	
Plasma Me	other			57863	94332	360882
Cytoplasm	other			800		
Plasma Me	other			10487	12331	64185
Plasma Me	other	disease progression		10486	67252	116653
Nucleus	other			22794	192160	259170
Cytoplasm	other	unspecified applicatio		845	12373	29209
Plasma Me	other			858	12390	363425
Cytoplasm	other			112464	109042	85332
Cytoplasm	enzyme			873	12408	29224|102556347
Cytoplasm	other			57639	75172	499980
Extracellul	cytokine	diagnosis,e	CNTO 888	6347	20293	287562
Extracellul	cytokine	disease progression,u		6363	24047|659	362506
Extracellul	cytokine		CM-101	6369	56221	288593

Extracellul	growth factor			4856	18133	81526
Nucleus	other			23582	17151	362201
Plasma Me	transmemb	unspecifie	6MW3211	961	16423	29364
Plasma Me	other		SGN-CD48	962	12506	245962
Plasma Me	transmembrane recep		blinatumor	915	12500	25710
Plasma Me	transmembrane recep		blinatumor	916	12501	315609
Plasma Me	transmemb	unspecified applicatio		973	12518	1.01E+08
Other	other			91368	52626	287278
Cytoplasm	enzyme			1036	12583	81718
Cytoplasm	enzyme			8760	110911	114101
Cytoplasm	enzyme			57214	80982	308797
Cytoplasm	enzyme			1066	104158	113902
Extracellul	other			1073	12632	366624
Extracellul	enzyme			1117		
Cytoplasm	enzyme			79586	74241	316533
Nucleus	transcription regulator			4261	12265	85483
Other	enzyme			440836	70113	500917
Plasma Me	transmembrane receptor			29121		312745
Plasma Me	other			26253	56619	450223
Plasma Me	ion channel			25932	29876	83718
Cytoplasm	other			1264	12797	65204
Extracellul	other		collagenas	7373	12818	314981
Extracellul	other		collagenas	1295	12837	304021
Cytoplasm	peptidase			51138	26891	360915
Nucleus	other			10920	108679	363283
Cytoplasm	other			11151	12721	155151
Cytoplasm	enzyme			1351	12868	171335
Cytoplasm	other			79974	214642	500046
Extracellul	peptidase			10404	54381	58952
Nucleus	other			29894	94230	366952
Cytoplasm	other			83690	83691	316482
Extracellul	other			55118	72832	171438
Cytoplasm	enzyme			1429	12972	362061
Cytoplasm	other			27254	105859	266600
Extracellul	other	diagnosis		1474	73720	171096
Extracellul	other			8530	13011	296257
Other	other					
Other	other					
Other	other					
Cytoplasm	peptidase			8722	56464	361704
Extracellul	cytokine	efficacy,unspecified a		10563	55985	498335
Extracellul	cytokine	diagnosis,disease pro		58191	66102	497942
Cytoplasm	enzyme			79901	73649	295669
Cytoplasm	enzyme			1537	66445	300047
Cytoplasm	enzyme	diagnosis,efficacy,pro		1545	13078	25426
Cytoplasm	enzyme		betametha	1595	13121	25427
Cytoplasm	other	unspecified applicatio		9267	19157	116691
Cytoplasm	other			27128	72318	500906
Cytoplasm	other			54987	74098	298384
Other	other			26528		
Extracellul	other					
Cytoplasm	other					

Cytoplasm	enzyme		
Extracellul	enzyme		
Extracellul	cytokine		
Cytoplasm	kinase	diagnosis	
Nucleus	other		
Cytoplasm	transporter		
Plasma Me	other		
Extracellul	other		
Cytoplasm	other		
Cytoplasm	phosphatase		
Cytoplasm	other		
Extracellul	other		
Extracellul	other		
Extracellul	other	diagnosis,p	parsatuzur
Cytoplasm	enzyme	prognosis	
Cytoplasm	translation regulator		
Nucleus	transcription regulator		
Plasma Me	other		
Nucleus	other		
Plasma Me	kinase		AZ126728
Cytoplasm	enzyme		
Cytoplasm	transporter	disease progression	
Plasma Me	enzyme	unspecified applicatio	
Other	other		
Other	other		
Other	other		
Other	other		
Nucleus	enzyme		
Cytoplasm	other		
Extracellul	other	efficacy	
Cytoplasm	other	disease progression	
Cytoplasm	other		
Extracellul	growth factor		
Extracellul	peptidase		
Nucleus	transcription regulator		
Cytoplasm	other	unspecified applicatio	
Plasma Me	other		
Cytoplasm	enzyme		
Nucleus	transcription regulator		
Nucleus	transcription regulator		
Extracellul	other		
Cytoplasm	enzyme	unspecified applicatio	
Cytoplasm	enzyme	diagnosis,unspecified	
Plasma Me	G-protein coupled rec		vantictuma
Cytoplasm	other	unspecified applicatio	
Extracellul	other		
Cytoplasm	other		
Cytoplasm	enzyme	efficacy	telaglenast
Cytoplasm	enzyme		
Cytoplasm	enzyme		
Plasma Me	enzyme		
Plasma Me	enzyme		

Cytoplasm	enzyme		
Cytoplasm	other		
Plasma Me	G-protein coupled receptor		
Cytoplasm	other		
Nucleus	other		
Cytoplasm	enzyme		isosorbide
Cytoplasm	enzyme		isosorbide
Cytoplasm	other		
Nucleus	other		
Nucleus	other		
Nucleus	other		
Plasma Me	transmembrane receptor		
Nucleus	transcription regulator		belinostat,l
Nucleus	transcription regulator		belinostat,|
Nucleus	transcription regulator		
Cytoplasm	other		
Cytoplasm	kinase		
Extracellul	other		
Nucleus	other		
Nucleus	other		
Nucleus	enzyme		
Cytoplasm	other		
Cytoplasm	other		
Cytoplasm	enzyme		diclofenac/
Nucleus	transcription regulator		
Cytoplasm	other	unspecified applicatio	
Plasma Me	other		
Extracellul	other	diagnosis,disease pro	
Extracellul	other		
Extracellul	other		
Extracellul	transporter		
Plasma Me	other		
Extracellul	cytokine	diagnosis,(	anti-IL-6 m
Plasma Me	transmemt	prognosis	
Extracellul	cytokine	efficacy	rilonacept
Plasma Me	transmemt	unspecifiec	aldesleukir
Plasma Me	transmemt	response t	corticoster
Plasma Me	phosphatase		
Cytoplasm	other		
Plasma Me	other		
Plasma Me	other		
Plasma Me	other	prognosis	
Plasma Me	other		
Plasma Me	other	diagnosis	cilengitide
Nucleus	enzyme		
Extracellul	other		
Nucleus	transcription regulator		
Nucleus	transcription regulator		
Plasma Me	ion channel		
Plasma Me	ion channel		betametha
Cytoplasm	other		
Other	other		

Nucleus	transcription regulator		
Cytoplasm	other		
Other	other		
Extracellul	enzyme		
Extracellul	other		
Other	other		
Other	other		
Nucleus	other		
Cytoplasm	enzyme		
Cytoplasm	other		
Plasma Me	G-protein coupled receptor		
Plasma Me	transmembrane recep		IO-108,JTX
Other	other	unspecified applicatio	
Plasma Me	G-protein coupled receptor		
Cytoplasm	other		
Extracellul	cytokine	safety,unspecified app	
Extracellul	other		
Cytoplasm	other		
Plasma Me	enzyme		
Cytoplasm	enzyme		benzpheta
Cytoplasm	other	efficacy	
Cytoplasm	kinase		K-252,SM1
Cytoplasm	other		
Plasma Me	other	diagnosis,prognosis,u	
Nucleus	transcription regulator		
Nucleus	enzyme		
Extracellul	other		
Extracellul	other		
Cytoplasm	enzyme		
Cytoplasm	enzyme	diagnosis,efficacy	
Cytoplasm	other		
Plasma Me	other		
Cytoplasm	other		
Plasma Me	G-protein coupled receptor		
Cytoplasm	other		
Cytoplasm	other		
Cytoplasm	other		
Cytoplasm	enzyme		
Other	other	diagnosis	
Cytoplasm	other		
Cytoplasm	enzyme		
Nucleus	transcription regulator		
Extracellul	cytokine		
Cytoplasm	enzyme	unspecified applicatio	
Cytoplasm	other		
Cytoplasm	enzyme		
Cytoplasm	other		
Nucleus	other		
Cytoplasm	other		
Extracellul	other		
Nucleus	enzyme		
Other	other		

Cytoplasm	kinase		
Nucleus	transcription regulator		
Cytoplasm	enzyme		
Cytoplasm	other		
Cytoplasm	transporter		
Nucleus	kinase		
Plasma Me	transmembrane receptor		
Cytoplasm	other		
Plasma Me	transporter		
Nucleus	peptidase		
Plasma Me	other		
Cytoplasm	enzyme	diagnosis,unspecified	
Extracellul	other		
Extracellul	other		
Cytoplasm	enzyme		
Cytoplasm	enzyme	diagnosis	eflornithine
Extracellul	growth factor		
Extracellul	other		
Extracellul	other		
Cytoplasm	enzyme		
Plasma Me	enzyme		
Extracellul	other		
Cytoplasm	enzyme		
Other	other		
Cytoplasm	enzyme		
Cytoplasm	enzyme		amrinone,a
Extracellul	cytokine		
Cytoplasm	enzyme		
Cytoplasm	enzyme		
Cytoplasm	enzyme		
Nucleus	transporter		
Cytoplasm	enzyme		
Cytoplasm	other		
Nucleus	other		
Cytoplasm	enzyme		
Nucleus	kinase		AZD-1208,
Plasma Me	ion channel		
Cytoplasm	other		
Other	other		
Cytoplasm	other		
Plasma Me	enzyme		
Plasma Me	other		CSR02-Fa
Other	other		
Nucleus	enzyme		
Nucleus	other		
Nucleus	other		
Cytoplasm	phosphatase		
Cytoplasm	other		
Nucleus	transcription regulator		
Cytoplasm	kinase		
Plasma Me	other		
Extracellul	other	diagnosis	

Nucleus	other		
Nucleus	transcription regulator		
Cytoplasm	other	diagnosis,disease pro	
Cytoplasm	peptidase		
Cytoplasm	enzyme	efficacy,unspecified a	
Cytoplasm	kinase		conteltinib,
Nucleus	phosphatase		
Other	other		
Cytoplasm	enzyme		
Other	other		
Cytoplasm	enzyme		
Cytoplasm	enzyme		
Cytoplasm	enzyme		
Cytoplasm	other		
Cytoplasm	other		
Cytoplasm	other		
Cytoplasm	other		
Cytoplasm	other		
Other	enzyme		
Plasma Me	enzyme		
Cytoplasm	other		
Cytoplasm	other		
Nucleus	enzyme		
Other	other		
Plasma Me	enzyme		
Cytoplasm	enzyme		AGS-16M1
Nucleus	enzyme		
Cytoplasm	enzyme		
Cytoplasm	enzyme		
Cytoplasm	kinase		belumosuc
Other	other		
Other	other		
Nucleus	other		
Cytoplasm	enzyme		
Cytoplasm	other		
Cytoplasm	other		
Nucleus	kinase		BI-D1870,I
Cytoplasm	enzyme		
Cytoplasm	enzyme		
Other	other		
Nucleus	other		
Nucleus	enzyme		
Nucleus	ligand-dependent nuc		acitretin,ali
Cytoplasm	other	diagnosis,efficacy,uns	
Cytoplasm	other	diagnosis,unspecified	
Plasma Me	G-protein coupled rec		etrasimod,
Other	other		
Other	transcription regulator		
Plasma Me	transmembrane receptor		
Cytoplasm	enzyme		
Cytoplasm	transporter		
Cytoplasm	other		

Cytoplasm	other		
Cytoplasm	transcription regulator		
Cytoplasm	transporter		
Plasma Me	transmemb	diagnosis	
Plasma Me	other		
Plasma Me	other		
Cytoplasm	other		
Cytoplasm	other		
Cytoplasm	other		
Plasma Me	transporter		
Cytoplasm	transporter		clodronic a
Plasma Me	transporter	diagnosis,e	alogliptin/Il
Plasma Me	transporter		
Extracellul	other		
Other	other		
Other	other		
Cytoplasm	phosphata	diagnosis,efficacy	
Cytoplasm	enzyme	unspecified	tofersen
Plasma Me	G-protein coupled rec		TH1902
Nucleus	transcription regulator		
Cytoplasm	other		
Cytoplasm	kinase	diagnosis,e	(2R,4S)-2-
Other	other		
Nucleus	transcription regulator		
Nucleus	enzyme		
Cytoplasm	other		
Cytoplasm	enzyme		
Cytoplasm	enzyme		
Plasma Me	transporter		FP-1305
Extracellul	other		
Cytoplasm	kinase		
Cytoplasm	other		
Plasma Me	transporter		
Plasma Me	transporter		
Nucleus	other		
Other	other		
Plasma Me	other		
Other	other		
Cytoplasm	other		
Other	other		
Cytoplasm	transporter	unspecified applicatio	
Plasma Me	G-protein coupled rec		ifetroban,ri
Other	other		
Nucleus	other		
Plasma Me	enzyme		
Cytoplasm	enzyme		
Plasma Me	other		
Nucleus	transcription regulator		
Extracellul	transporter		
Plasma Me	G-protein c	unspecified applicatio	
Other	transporter		
Plasma Me	other		

Cytoplasm	other		
Other	other		
Cytoplasm	transporter		
Cytoplasm	transporter		
Extracellul	other		
Extracellul	cytokine	efficacy	ABBV-621
Cytoplasm	transporter		
Nucleus	enzyme		alemtuzum
Cytoplasm	other	diagnosis	
Extracellul	other		
Cytoplasm	enzyme		
Cytoplasm	kinase		
Nucleus	enzyme		
Extracellul	enzyme		
Cytoplasm	transporter		
Plasma Me	ion channel		
Extracellul	other		
Plasma Me	other		
Cytoplasm	transmembrane recep		alprazolam
Cytoplasm	other		
Cytoplasm	other		6-mercapto
Extracellul	other		
Extracellul	growth fac	diagnosis,e	tipiracil
Cytoplasm	enzyme		
Cytoplasm	enzyme		
Cytoplasm	enzyme		
Cytoplasm	enzyme		
Nucleus	other		
Cytoplasm	other		
Cytoplasm	enzyme		
Plasma Me	other	unspecified applicatio	
Cytoplasm	ion channel		
Nucleus	other		
Cytoplasm	other		
Plasma Me	other		CA-170,CI
Extracellul	other	diagnosis,e	caplacizum
Cytoplasm	other		
Cytoplasm	other		
Nucleus	transcription regulator		
Nucleus	transcription regulator		verteporfin
Nucleus	other		
Cytoplasm	other		
Extracellul	other		
Cytoplasm	other		
Other	other		
Nucleus	transcription regulator		
Nucleus	transcription regulator		
Nucleus	transcription regulator		
Other	other		
